# Linking functional and structural brain organisation with behaviour in autism: a multimodal EU-AIMS Longitudinal European Autism Project (LEAP) study

**DOI:** 10.1186/s13229-023-00564-3

**Published:** 2023-08-31

**Authors:** Lennart M. Oblong, Alberto Llera, Ting Mei, Koen Haak, Christina Isakoglou, Dorothea L. Floris, Sarah Durston, Carolin Moessnang, Tobias Banaschewski, Simon Baron-Cohen, Eva Loth, Flavio Dell’Acqua, Tony Charman, Declan G. M. Murphy, Christine Ecker, Jan K. Buitelaar, Christian F. Beckmann, The EU-AIMS LEAP Group, Jumana Ahmad, Sara Ambrosino, Bonnie Auyeung, Tobias Banaschewski, Simon Baron-Cohen, Sarah Baumeister, Christian F. Beckmann, Sven Bölte, Thomas Bourgeron, Carsten Bours, Michael Brammer, Daniel Brandeis, Claudia Brogna, Yvette de Bruijn, Jan K. Buitelaar, Bhismadev Chakrabarti, Tony Charman, Ineke Cornelissen, Daisy Crawley, Flavio Dell’Acqua, Guillaume Dumas, Sarah Durston, Christine Ecker, Jessica Faulkner, Vincent Frouin, Pilar Garcés, David Goyard, Lindsay Ham, Hannah Hayward, Joerg Hipp, Rosemary J. Holt, Mark H. Johnson, Emily J. H. Jones, Prantik Kundu, Meng-Chuan Lai, Xavier Liogier D’ardhuy, Michael V. Lombardo, Eva Loth, David J. Lythgoe, René Mandl, Andre Marquand, Luke Mason, Maarten Mennes, Andreas Meyer-Lindenberg, Carolin Moessnang, Nico Mueller, Declan G. M. Murphy, Bethany Oakley, Laurence O’Dwyer, Marianne Oldehinkel, Bob Oranje, Gahan Pandina, Antonio M. Persico, Jack Price, Annika Rausch, Barbara Ruggeri, Amber N. V. Ruigrok, Jessica Sabet, Roberto Sacco, Antonia San Jóse Cáceres, Emily Simonoff, Will Spooren, Julian Tillmann, Roberto Toro, Heike Tost, Jack Waldman, Steve C. R. Williams, Caroline Wooldridge, Iva Ilioska, Ting Mei, Marcel P. Zwiers, Natalie J. Forde

**Affiliations:** 1 Department of Cognitive Neuroscience, Donders Institute for Brain, Cognition and Behaviour, Radboud University Nijmegen Medical Centre, Kapittelweg 29, 6525 EN Nijmegen, The Netherlands; 2 Karakter Child and Adolescent Psychiatry University Centre, Nijmegen, The Netherlands; 3 Department of Psychology, Institute of Psychiatry, Psychology and Neuroscience, King’s College London, London, UK; 4 Department of Psychiatry, Brain Center Rudolf Magnus, University Medical Center Utrecht, Utrecht, The Netherlands; 5 Department of Psychiatry and Psychotherapy, Central Institute of Mental Health, Medical Faculty Mannheim, University of Heidelberg, Mannheim, Germany; 6 Department of Child and Adolescent Psychiatry, Central Institute of Mental Health, Medical Faculty Mannheim, University of Heidelberg, Mannheim, Germany; 7 Autism Research Centre, Department of Psychiatry, University of Cambridge, Cambridge, UK; 8 Department of Forensic and Neurodevelopmental Sciences, Institute of Psychiatry, Psychology and Neuroscience, King’s College, London, UK; 9 Department of Child and Adolescent Psychiatry, University Hospital, Goethe University, Frankfurt am Main, Germany; 10 Methods of Plasticity Research, Department of Psychology, University of Zurich, Zurich, Switzerland

**Keywords:** Linked ICA, Autism spectrum disorder, Brain-behaviour associations

## Abstract

Neuroimaging analyses of brain structure and function in autism have typically been conducted in isolation, missing the sensitivity gains of linking data across modalities. Here we focus on the integration of structural and functional organisational properties of brain regions. We aim to identify novel brain-organisation phenotypes of autism. We utilised multimodal MRI (T1-, diffusion-weighted and resting state functional), behavioural and clinical data from the EU AIMS Longitudinal European Autism Project (LEAP) from autistic (*n* = 206) and non-autistic (*n* = 196) participants. Of these, 97 had data from 2 timepoints resulting in a total scan number of 466. Grey matter density maps, probabilistic tractography connectivity matrices and connectopic maps were extracted from respective MRI modalities and were then integrated with Linked Independent Component Analysis. Linear mixed-effects models were used to evaluate the relationship between components and group while accounting for covariates and non-independence of participants with longitudinal data. Additional models were run to investigate associations with dimensional measures of behaviour. We identified one component that differed significantly between groups (coefficient = 0.33, *p*_adj_ = 0.02). This was driven (99%) by variance of the right fusiform gyrus connectopic map 2. While there were multiple nominal (uncorrected *p* < 0.05) associations with behavioural measures, none were significant following multiple comparison correction. Our analysis considered the relative contributions of both structural and functional brain phenotypes simultaneously, finding that functional phenotypes drive associations with autism. These findings expanded on previous unimodal studies by revealing the topographic organisation of functional connectivity patterns specific to autism and warrant further investigation.

**Supplementary Information:**

The online version contains supplementary material available at 10.1186/s13229-023-00564-3.

## Background

Autism spectrum disorders (autism) are characterised by difficulty with communication and social interaction and repeated stereotyped behaviours and/or altered sensory processing [1]. Neuroimaging studies of autism have furthered our understanding of its neurobiology; however, the majority of studies thus far have been limited in their ability to identify valid and reliable biomarkers. Classical neuroimaging studies have used case–control designs, together with a typical focus on small samples and single data modalities. This approach potentially obfuscates biomarker identification since individual variability is masked by focusing only on between group effects, thereby ignoring group heterogeneity. Furthermore, sensitivity to associations is lacking due to the fragmented analytical approach of single modality analysis.

Recent significant progress in neuroimaging studies has been made by addressing and utilising the heterogeneity of autism—focusing on dimensional measures of behaviour that cut across diagnostic boundaries [2–7]—and by using advanced methodologies that account for diverse patterns of variance across the brain rather than focal deviations [3, 8–13]. Three of these latter studies [3, 8, 9] revealed covariation patterns of grey matter density and deviations in cortical thickness, associated with autism in the same sample considered in the present paper. As well as primary univariate findings of association with autism, these papers also revealed complex associations between all isolated imaging phenotypes and multiple continuous clinical measures as determined in a multivariate analysis conducted with canonical correlation analysis (CCA). These papers show the potential of data-driven approaches to leverage the heterogeneity of autism in pursuit of biomarkers.

Moreover, it has been demonstrated that moving from separate unimodal MRI analysis to integrated multimodal analysis with linked independent component analysis (LICA) generates brain phenotypes that strongly relate to demographic and behavioural data, far exceeding the potential of any individual unimodal approach seen to date [14, 15]. The assumption with multimodal approaches is that underlying pathophysiological processes are reflected in multiple aspects of neurobiology, such that different indices of these biological measures can be used to achieve a joint integrated picture of these processes. Thus, by integrating information from multiple sources we gain increased sensitivity to detect associations with behaviour. Specifically, LICA, an extension of traditional ICA, decomposes such multimodal data to generate a set of spatial maps, subject-specific and modality-specific contributions for each independent component (IC). Recently, this method has been successfully implemented in attention deficit hyperactivity disorder (ADHD) research to identify novel brain phenotypes associated with ADHD severity [16] and in autism research to integrate clinical and event related potential (ERP) data, which identified early neuronal processes that predicted clinical outcome [17]. There are also a small number of autism studies that integrate different MRI modalities using LICA. However, one of these utilised LICA for visualisation purposes only [18] while the other 2 investigated the LICA subject courses in analysis. Both these studies integrated voxel based morphology (VBM) grey matter density maps and diffusion tensor imaging (DTI) metrics. Itahashi and colleagues identified one component that showed covariations in both grey matter and white matter morphology associated with autism diagnosis [19] in a relatively small sample (*n* = 92 total) of adult males, all with an IQ of > 80. More recently a second study from Mei and colleagues [20] analysed a larger group with broader spread across IQ, age and sex, and identified one multimodal pattern associated with autism diagnosis [20]. The grey and white matter covariation patterns differed between these two papers, likely due to the differences in the sample demographics.

Previous implementations of MRI-based LICA, mentioned above, used traditional unimodal data extraction techniques. Here we attempted to improve upon previous approaches by incorporating more analytically advanced initial processing steps developed in recent years, leading to more biologically plausible unimodal representations of the brain prior to integration. Using these advanced unimodal feature extraction methods may provide more accurate characterisation of the fine-grained features of the brain. For instance, we know that there is behaviourally relevant functional organisation within brain regions and potential multiplicity of these regions [21–25]. Connectopic mapping is able to characterise within region of interest (ROI) variations in functional connectivity while simultaneously dealing with potential functional multiplicity [21–25], substantially improving upon traditionally used network-based functional connectivity metrics. We therefore use connectopic mapping to identify functional connectopies (gradients) within our resting-state functional MRI (fMRI) data. Additionally, structural organisation in the brain can be modelled from diffusion MRI (dMRI) data with non-tensor based tractography, providing a more biologically plausible representation of the white matter than traditional tensor-based methods which fail to model complex fibre architectures [26]). We therefore implement a non-tensor based method to better address this complexity. Further, implementing probabilistic tractography from each voxel within an ROI allows us to probe the potential spatial organisations of structural connectivity. We hypothesised that these advanced approaches to unimodal feature extraction coupled with multimodal integration via LICA and implemented on a large sample would provide us with additional sensitivity to detect brain-behaviour relationships relevant to autism.

Here we test if the implementation of improved modelling of functional and structural connectivity may yield a more fine-grained characterisation of neurobiological variation in autism, and explore if an integrated multimodal approach can identify new, MRI-based autism-related brain phenotypes. Ultimately, the identification of more fine-grained, multimodal autism related phenotypes could enhance our understanding of how autism is represented in the brain across modality boundaries, and additionally increase sensitivity to detect associations with behavioural measures of autism. This could improve the early detection of autism and might lead to novel avenues of intervention down the line.

## Methods

### Participants

Data from the European Autism Interventions—A Multicentre Study for Developing New Medications (EU-AIMS) Longitudinal European Autism Project (LEAP) [27] was used. This is a large European multicentre study focusing on identifying and validating biomarkers for autism. In total, six centres are involved; however, due to limited availability of high quality MRI data in all modalities, one site was excluded. The 5 remaining sites were: Institute of Psychiatry, Psychology and Neuroscience, King’s College London, United Kingdom; Radboud University Medical Centre, Nijmegen, the Netherlands; Central Institute of Mental Health, Mannheim, Germany; Cambridge University, Cambridge, UK and University Medical Centre Utrecht, the Netherlands. Local ethics committees in each participating centre approved the study and written informed consent was provided by all participants and/or their legal guardians (for those < 16/18 years old [country dependent] or legally incapacitated). Details of the project have been outlined elsewhere [27], but briefly; participants with and without autism underwent clinical, cognitive and MRI assessment at multiple timepoints.

Only participants with all of the required modalities (T1-weighted, resting state-fMRI and diffusion weighted imaging [DWI] at any one time point), of sufficient quality (see Additional file 1 for quality assessment details), were included in this study. This resulted in 206 autism and 196 neurotypical (NT) participants. 97 of these had good quality data of all modalities available at 2 timepoints. See Table 1 and Additional file 1: Table S2 for demographic and clinical information. This sample was not sufficient for longitudinal analysis. Data from both timepoints is used regardless, as it increases our sample size by an additional 97 scans, giving us more statistical power.

**Table 1:** Demographics of individuals at first or only time point

	Autism	Control	Test statistic	*p* value
*N*	206	196		
Sex *m*:*f*	147:59	124:72	*χ*^2^ = 2.6	0.1
Age mean (SD), years	17.8 (5.2)	17.3 (5.2)	*χ*^2^ = 393	0.5
IQ mean (SD)	101 (20)	105 (18)	*χ*^2^ = 188	0.05
Timepoint *t*1:*t*2	166:40	149:47	*χ*^2^ = 1	0.3
**Site**				
Cambridge	14	14	*χ*^2^ = 9	0.06
KCL	74	67	*χ*^2^ = 9	0.06
Mannheim	24	36	*χ*^2^ = 9	0.06
Nijmegen	81	57	*χ*^2^ = 9	0.06
Utrecht	13	22	*χ*^2^ = 9	0.06

### Clinical assessment

All clinical assessments that were utilised were collected at both wave 1 and wave 2. Participants were included in the autism group if they had a clinical diagnosis according to the DSM-IV or DSM-5 criteria. Within the autism group, symptoms in the domains of social affect and restricted repetitive behaviours (RRB) were assessed with the Autism Diagnostic Observational Schedule 2 (ADOS-2) [28]. To assess symptoms in adaptive behaviour impairment the Vineland adaptive behaviour scale (VABS) was used [29]. Specifically, impairments in socialisation, communication, daily living skills and motor skills were determined with the VABS scale. In both autistic and non-autistic participants we further assessed autistic symptoms and repetitive and rigid behaviours with the Social Responsiveness Scale 2nd Edition (SRS) [30] and the Repetitive Behaviour Scale-Revised (RBS) [31], respectively. Finally, sensory processing was assessed with the Short Sensory Profile (SSP) [32]. Given the high rate of comorbidity of ADHD in autism we assessed ADHD symptoms with the DSM-5 rating scale (parent report or self-report when parent report was unavailable). Full scale IQ (fsIQ) was estimated from 4 subtests of the Wechsler Abbreviated Scales of Intelligence (WASI) or Wechsler Adult Intelligence Scale (WAIS)/Wechsler Intelligence Scale for Children (WISC) as appropriate and available in local languages during participants' first visit (LEAP wave 1).

### MRI data acquisition

All participants were scanned on 3T MRI scanners at both time points. T1-, functional and diffusion- weighted MRI data were acquired across five sites using largely the same scanner parameters. The details on the individual scanning parameters and quality control and pre-processing procedures are described in the Additional file 1 (Sects. 2–5). See Additional file 1: Table S3 for scan parameters.

### Unimodal feature extraction

**Fig. 1:**
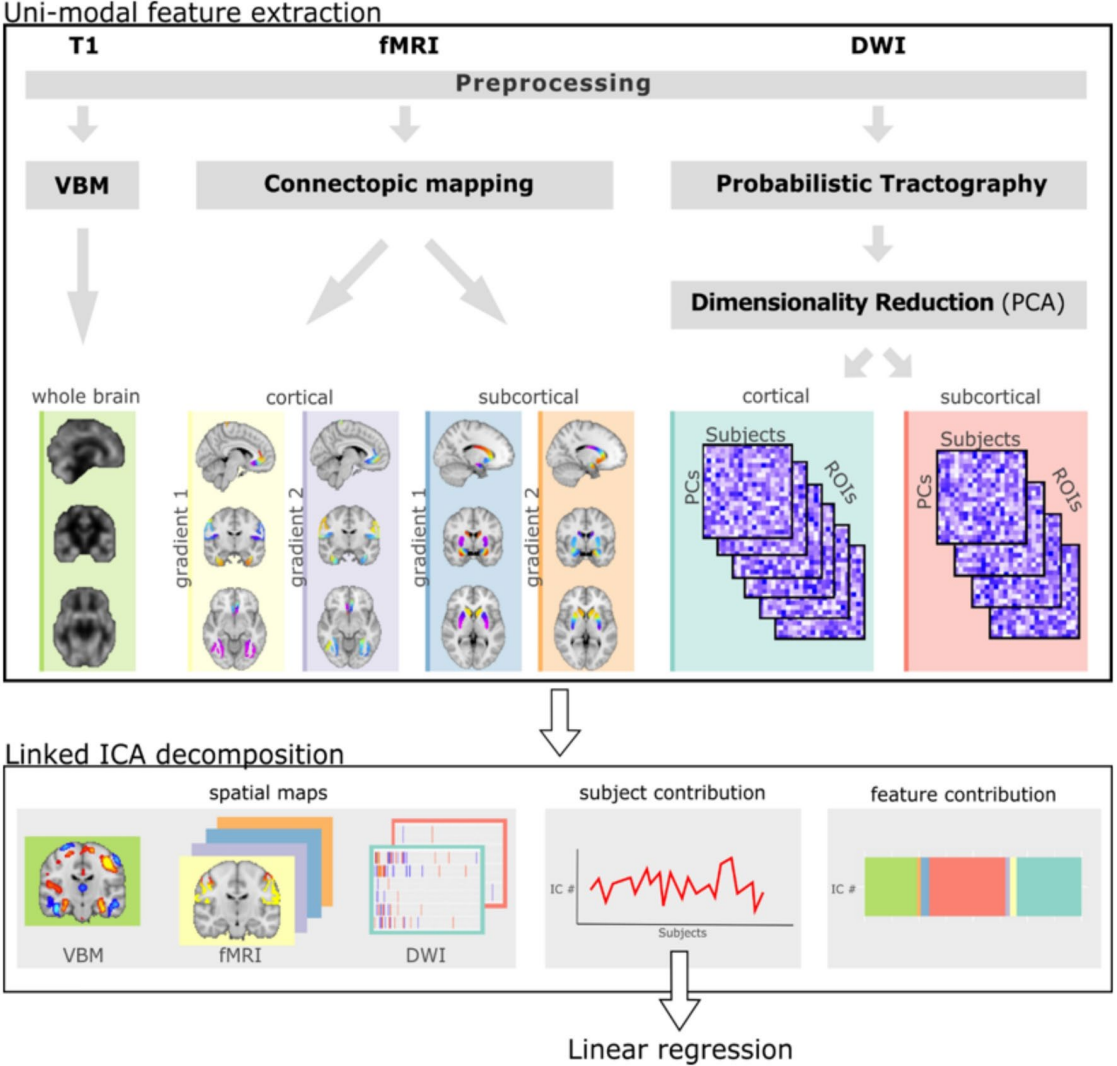
Overview of methods. This figure shows a conceptual overview of the analysis pipeline. The upper portion shows the unimodal feature extraction prior to integration. The bottom part shows the outputs of multimodal integration using LICA. DWI—diffusion weighted imaging, T1—T1-weighted magnetic resonance imaging, fMRI—functional magnetic resonance imaging, PCA—principal component analysis, IC—independent component, ICA—independent component analysis, VBM—voxel based morphometry

The unimodal features we use for multimodal integration with LICA are derived from advanced MRI data processing pipelines, further described in the sections below. Briefly, we derive whole-brain grey matter density maps from T1-weighted images, connectopic maps from rs-fMRI scans and white matter connectivity measures from probabilistic tractography. See Fig. 1 for an overview of the unimodal feature extraction and multimodal integration.

### T1-weighted data

T1-weighted MRI data were processed with the Voxel Based Morphometry (VBM) pipeline in the computational anatomy toolbox (CAT12: http://www.neuro.uni-jena.de/cat/) in statistical parametric mapping software (SPM12: Wellcome Department of Imaging Neuroscience, London, UK). T1-weighted images were automatically segmented into grey matter, white matter and cerebrospinal fluid and affine registered to the MNI template. Resulting segmented grey matter maps were then used to generate a study-specific template (excluding longitudinal data) and registered to MNI space via a high-dimensional, nonlinear diffeomorphic registration algorithm (DARTEL) [33]. All data were then processed using this template. A Jacobian modulation step was included using the flow fields to preserve voxel-wise information on local tissue volume. Images were smoothed with a 4 mm full-width half-max (FWHM) isotropic Gaussian kernel and downsampled (for computational reasons) to 2 mm isotropic voxel dimension.

### ROI selection for DWI and RS analysis

Due to computational restrictions, analysis had to be limited to select ROIs. We chose to focus on regions that have previously been implicated in autism such as the amygdala, striatum, post central gyrus, fusiform gyrus and anterior cingulate cortex [34, 35]. Subcortical ROIs were isolated from the Harvard–Oxford atlas while cortical ROIs were isolated from the Desikan-Kiliany atlas [36]. The striatum ROI was formed by combining the Nucleus Accumbens, Putamen and Caudate structures per hemisphere as per previous connectopic mapping studies [22].

### Diffusion weighted imaging (DWI)

DWI data were extensively preprocessed to correct for imaging and movement artefacts, see Additional file 1 for details. Data were then processed with Bayesian Estimation of Diffusion Parameters Obtained using Sampling Techniques (BEDPOSTX) and probabilistic tractography (ProbtrackX) [37]. ProbtrackX was performed with seeding from select ROIs and a list of target atlas ROIs to generate a voxel by target ROI connectivity matrix for each seed ROI. The target list was composed of cortical regions from the multi-modal parcellation developed on the Human Connectome Project (HCP) data [38] and subcortical ROIs from the Harvard–Oxford atlas. A MNI 2 × 2 × 2 mm^3^ template was used for atlas regions to ensure spatial correspondence across subjects. Principal component analysis (PCA) was used to reduce the dimensionality of these data. Matrix rank-1 PCs were kept for analysis in LICA. Participant-by-PC matrices for the different cortical or subcortical seed ROIs were stacked to produce one cortical and one subcortical input for LICA.

### Resting state functional MRI (rs-fMRI)

Rs-fMRI data were preprocessed to account for movement and scanning artefacts as previously described [39] and outlined in the Additional file 1. On the preprocessed data we performed connectopic mapping to generate 3 connectopic maps of each ROI determined by their functional connectivity to the rest of the cortex [21]. Reference gradients were produced by averaging gradients from 20 subjects from the Human Connectome Project [40]. All subject gradients were then checked against these to ensure consistent ordering and direction (i.e. not flipped). Gradients 1 and 2 were selected for further analysis. Gradient 1 for each cortical or subcortical ROI was combined into single spatial maps, while gradient 2 was similarly combined into others, thereby producing 4 inputs for LICA; cortical G1, cortical G2, subcortical G1 and subcortical G2.

### Multimodal integration

LICA is an extension to ICA that allows for the integration of multi-modal data linked through a shared mixing matrix [41]. For each independent component (IC) isolated the algorithm provides a set of spatial maps (one per original modality), a vector describing the contribution of each subject and finally a vector of the loading weights showing the contribution of each modality to that component. We use the vector containing the subject loadings per (multimodal) IC to investigate the relationship between the (multimodal) brain phenotypes and demographic/behavioural measures. Given our sample size, we generated 80 components for this analysis. We additionally calculated a multimodal index (MMI) per IC as previously described [16], indicating if a component was driven exclusively by one or multiple of the original data modalities.

### Statistical analysis

Statistical analyses and graph generation were all performed in R (V3.5.1, [42]). Building on previous work that found group differences in structural brain phenotypes between autistic and non-autistic participants in the same cohort [3, 20] we first used linear mixed effects models to test the association between our imaging derived brain phenotypes and autism diagnosis while accounting for age, sex, scan site and time point of collection. Non-independence of some participants (longitudinal data) were accounted for with subject ID as a random factor. Analyses of diagnosis were False-Discovery Rate (FDR) corrected for the number of IC’s tested [43].

Similar models were then utilised to investigate continuous associations with ADOS total and its subscales and VABS scales within the autism group, and the SSP, RRB, SRS questionnaire data across the full sample. FDR [43] multiple comparison correction was used in these analyses.

Significant associations were further probed to see if they were robust to the inclusion of fsIQ in the model. Additionally, we tested the associations for interaction effects with site, sex, age and time point.

### Additional analysis

Data integration and statistical analyses were repeated in structural or functional only subsets of features to determine if combined or separate analysis yielded more promise.

As a post-hoc analysis we applied trend surface modelling (TSM) to the connectopic gradient of our main finding. Essentially, TSM breaks down a surface into fewer trend coefficients that reflect the general trends of the surface, here, of the connectopic gradient per subject [22, 44]. We applied model order (MO) of 3 to avoid overfitting. This yielded 9 coefficients which we tested for association with autism diagnosis using linear mixed effects models identical to those described above. Then, we applied Bonferroni correction as multiple comparison correction (MCC), adjusting for the number of coefficients analysed.

## Results

### Participants

The final cohort of participants consisted of *N* = 206 autistic and *N* = 196 non-autistic individuals. The groups did not differ significantly in terms of sex distribution, age or fsIQ across sites or collection wave (all *p* values > 0.05, Table 1). Longitudinal data was only available for 2 of the 5 sites (Nijmegen and Mannheim; Additional file 1: Table S2). The longitudinal cohort consisted of *N* = 51 autistic participants and *N* = 46 non-autistic participants. Similar to the cross-sectional cohort there were no significant differences between the groups in terms of sex, age, fsIQ or diagnostic distribution across sites (all *p* values > 0.09, Additional file 1: Table S2). Given the small amount of longitudinal data available in comparison with the whole sample and the short duration between timepoints we choose to analyse the whole sample together focussing on main effects of diagnosis and behaviour rather than longitudinal effects.

### Group associations

**Fig. 2:**
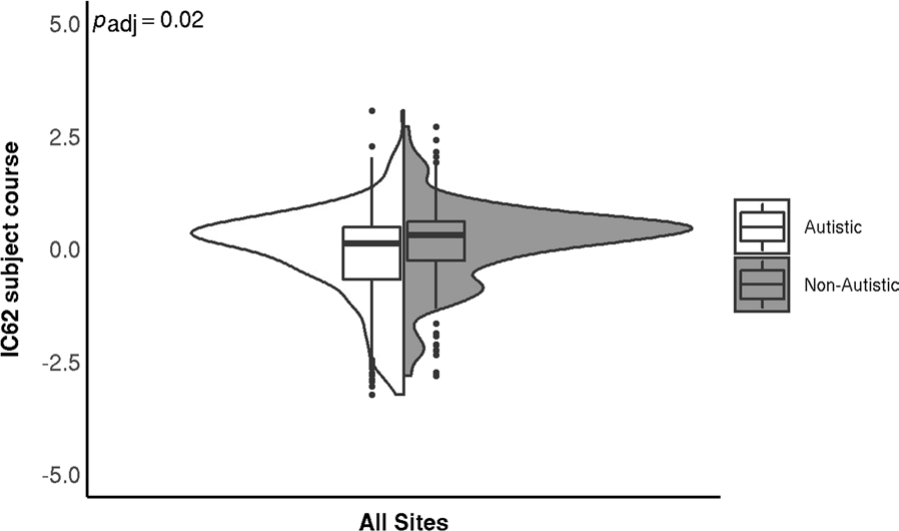
IC62 subject course per group. The violin plots show the distribution of the subject courses while the boxplots indicate the first and third quartile with the median denoted with a thick horizontal line. There was a significant main effect of diagnostic group. The FDR-adjusted *p* value is shown in the top left corner. There was no significant effect of site or diagnosis-by-site interaction

Our analysis yielded one significant association after MCC, between IC62 and autism diagnosis, where the contribution from the autistic group was lower than the non-autistic group (coefficient = 0.33, *p*_adj_ = 0.02; Fig. 2). This effect was robust to the addition of full-scale IQ (fsIQ) and no significant interactions were found between diagnosis and sex, age, site, timepoint or fsIQ (*p* values > 0.1; data plotted per site is shown in Additional file 1: Figure S3). Furthermore, as this component was almost solely driven by the functional data we tested for confounding effects of in-scanner motion, indexed as average framewise displacement (FD) during the resting state scan. We found our diagnosis effect was robust to its inclusion in the model (coefficient = 0.33, *p*_adj_ = 0.02) and there was no significant effect of FD on the subject course (*χ*^2^ = 0.19, *p* = 0.66).

**Fig. 3:**
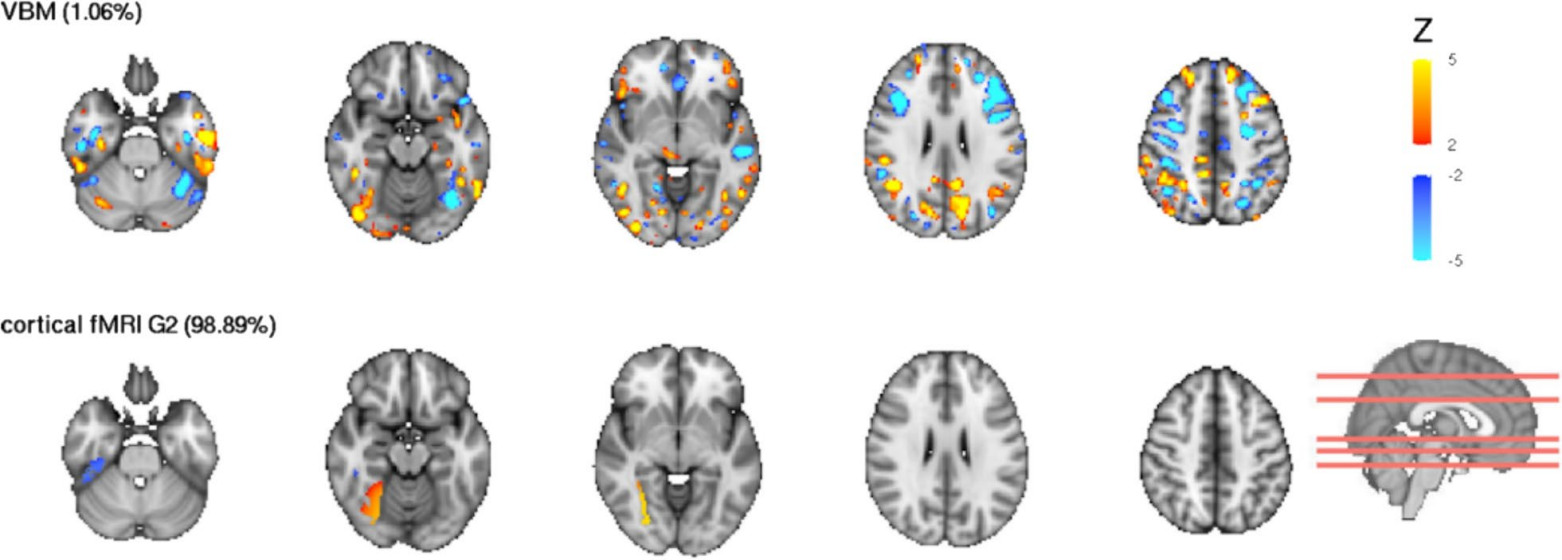
Spatial maps of IC62. Modality contributions of IC62 are shown. Modalities contributing < 1% are excluded from visualizstion. The scale represents the *Z*-score of spatial contribution within each feature. The red lines on the sagittal slice indicate the position of the axial slices displayed. VBM—voxel based morphology, fMRI G2—function MRI gradient 2. |*Z*|> 2 is shown

The cortical connectopic gradient 2 feature contributed mostly to this component (99%), localised in the fusiform gyrus of the right hemisphere (Fig. 3). The component additionally had a VBM contribution of 1% (Fig. 3), while the other modalities contributed close to 0%.

**Fig. 4:**
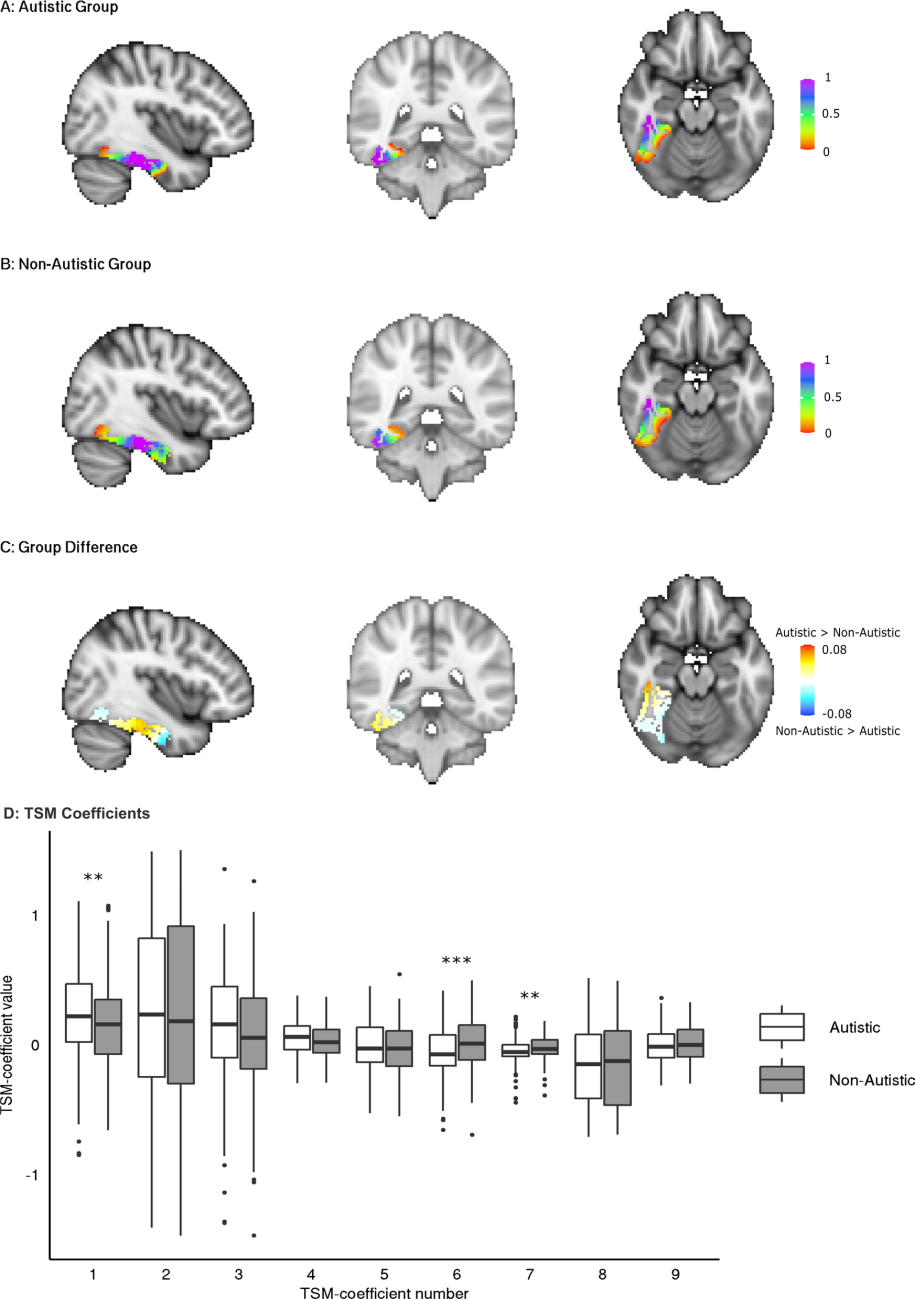
Group average connectopic maps of the right fusiform gradient 2 and TSM coefficients per group. **A** Shows the average gradient of the autistic group. **B** Shows the average gradient of the non-autistic group. The colour scale represents the similarity of functional connectivity between voxels and the rest of the brain, with similar colours representing a similar connectivity pattern. **C** Is the group difference map. Red indicates where the autistic group had higher connectivity gradient values compared to the non-autistic group. Blue indicates the opposite—where the autistic group had lower connectivity gradient values compared to the non-autistic group. **D** Shows the values of TSM-Coefficients as boxplots showing the first and third quartile with the median denoted with a thick horizontal line. The plots are split by group. Coefficients that are significantly different between groups post-MCC are denoted with asterisks. **p_adj_ ≤ 0.01, ***p_adj_ ≤ 0.001

To determine if we could detect group specific connectopic gradients we went on to visualise the average gradient for each group. We found that the group average gradient maps display the same general pattern in their connectopic maps. There is a medial–lateral primary axis to the gradient with the medial aspect extending to the posterior and anterior extremities of the structure. Notably, however, these group average gradient maps subtly visually differ (Fig. 4A, B). To highlight differences in the connectivity patterns, we calculated the difference map between the gradients by subtracting the non-autistic group average map from the autistic group average map (Fig. 4C). We see a slight shift, on average, in the spatial organisation of functional processing between groups. We further tested this by extracting TSM-coefficients of the right hemisphere fusiform gyrus for each subject, thereby reducing the dimensionality of the complex connectopic gradient to nine coefficients that reflect the general connectopic trend across the ROI. Using linear mixed effects models. We found three out of nine coefficients were significantly associated with diagnosis after correcting for multiple testing (TSM-coefficient 1: *coefficient* = − 0.11, *p*_adj_ = 0.008; TSM-coefficient 6: *coefficient* = 0.07, *p*_adj_ = 0.0004; TSM-coefficient 7: *coefficient* = 0.03, *p*_adj_ = 0.001; Fig. 4D). The other 6 TSM-coefficients were not significantly associated with diagnosis (*p*_adj_-values > 0.05). To determine the reproducibility of these associations we conducted a split-half analysis outlined in the Additional file 1. The outcome of this analysis showed that the same coefficients are associated with diagnosis in random halves of the same data.

### Behavioural associations

The present work found only nominal (uncorrected *p* < 0.05) associations between imaging derived phenotypes and dimensional clinical measures (ADOS, SRS, SSP, RBS, VABS), meaning that the associations are initially significant but do not survive MCC using FDR. See Additional file 1 for more information on these nominal results.

### Additional analyses

Structural only analysis revealed one multimodal component, IC42, that nominally related to group and many nominal behavioural associations. Functional only analysis also revealed multiple nominal associations, IC20 and IC55, with both group and behavioural measures. Details are presented in the Additional file 1. IC55 in particular showed very strong correlations with our main finding of IC62, in both the subject contributions (rho = 0.63, *p*_perm_ < 0.001) and the spatial maps (cortical gradient 2 *r* = 0.96).

## Discussion

In the present paper we aimed to improve upon previous approaches that investigated the complex neurobiology of autism. We implemented advanced unimodal feature extraction pipelines to focus on brain structural and functional organisation with the hope of producing imaging derived phenotypes that are more sensitive to the microstructural properties of the brain prior to multimodal integration. The increased sensitivity to subtle variations within ROIs allowed us to decompose across more fine-grained representations of unimodal brain features. We identified one component, IC62, as significantly associated with autism diagnosis after accounting for multiple testing. This component was mainly driven by the functional connectopic gradient 2, specifically localised to the right fusiform gyrus. This result was found to be robust to confounding effects of acquisition site and fsIQ. There were also multiple nominal associations of other ICs with diagnosis and/or behavioural measures relevant to autism but these did not survive MCC.

Our primary result identified one component, IC62, which is mainly driven by cortical gradient 2 in the fusiform gyrus and significantly associated with autism diagnosis. The localisation of this finding is in accordance with previous unimodal literature which identified decreased activation of the fusiform gyrus when autistic participants view faces [45, 46]. The fusiform gyrus has been shown to be highly involved in higher-order visual processing, specifically the perception of faces, object recognition and reading comprehension [47]. Face perception has furthermore been shown to be highly lateralized, with the right fusiform gyrus playing a larger role in distinguishing between face versus non-face [48]. Altered responses to emotions in facial expressions are among the key behavioural phenotypes associated with autism [1]). Based on structural connectivity patterns of the fusiform gyrus it was previously found that the fusiform gyrus can be clustered into 3 subregions (medial, lateral and anterior), with each subregion associated with a distinct functional connectivity pattern [49]. Due to the advanced unimodal feature extraction methods applied in this work our results are more sensitive to functional connectivity changes within the fusiform gyrus. Here our first gradient displayed a main axis in the anterior–posterior direction. While our second gradient, the one significantly associated here with autism diagnosis, displayed a medial–lateral organisation with the medial pattern extending anterior and posterior to the peripheral extents of the fusiform. Analysis of extracted TSM-coefficients from the right fusiform second gradient confirmed the association of topographically organised functional connectivity patterns with autism diagnosis. This expands on the results of previous unimodal studies by identifying a topographical organisation of functional connectivity within the fusiform gyrus associated with autism.

Across all associations found (including nominal), we see little shared variance between functional and structural modalities. Moreover, the majority of findings were driven by unimodal functional variation rather than structural. This implies that our functional data processing using connectopic mapping may be fruitful in providing insights into the neurobiology of autism, behaviours relevant to autism, and neurodevelopmental conditions more generally. While the integration of functional and structural data presented here did not provide specific insights into how function and structure together vary with behaviour, it notably allows us to simultaneously characterise phenotypes across structure and function, thereby directly testing their relative contributions. Thus, this approach meaningfully contributes to the ongoing structure–function debate. Llera and colleagues [15] found their structural measures captured the variance associated with multiple behavioural traits and the addition of functional connectivity data to the analysis contributed little in terms of variance explained. This is in contrast to our current findings which show that the functional data dominate components throughout the decomposition and provide the majority of behavioural associations. This contrast likely stems from the different methods of unimodal feature extraction used. Here we used connectopic mapping in contrast to functional network based connectivity measures previously utilised. These findings imply that inter-individual variance of network based connectivity measures can mostly be captured by variance in structure alone while connectopic maps show inter-individual variability independent of structure. While some of the extracted unimodal connectopic map based components may be noise related, the prominence of connectopic mapping in the ICs that are associated with autism and/or behaviours found here indicates there is potentially behavioural relevance captured. However, many of these findings were nominal and therefore require further investigation and validation.

Structural only analysis (see Additional file 1) revealed one component, IC42, that was nominally associated with the autism diagnosis. This component showed co-varying white matter organisation and grey matter density patterns (i.e. was multimodal). The component also relates to components reported previously, on partially overlapping samples, that investigated grey matter co-variation patterns in autism [3] and co-varying grey and white matter co-variation patterns [20]. However, our finding was not significant after multiple comparison correction, nor was it stable across sites (see Additional file 1 for details).

Functional only analysis (see Additional file 1) revealed two components, IC20 and IC55, that were significantly associated with autism diagnosis. The high correspondence between the subject course and the spatial map of IC55 to our main finding implies that we capture largely the same variance in the combined LICA and the functional data only LICA.

### Limitations

Our proxies of organisation for functional and structural data are based on different methodologies. Connectopic mapping is a nonlinear method which generates multiple functional topographic maps based on the connectivity of a region to the rest of the brain. We use a linear decomposition (PCA) on our probabilistic tractography data to address the sparsity of connectivity data before integration with LICA. Connectopic mapping of the tractography data [50] would also have been possible but is computationally resource heavy and selecting the number of gradients to retain would have been arbitrary given the scarcity of studies utilising this method to date. By using PCA we retained more of the variance of the data while still reducing the dimensionality. However, investigation of both structurally and functionally derived connectopic maps together is warranted. Second, for computational reasons we limited our analysis to select ROIs. These ROIs were selected based on their previous implication in autism. However, a brain-wide approach may have uncovered more associations and are encouraged in the future work. Finally, our need for all 3 imaging modalities of good quality to be available for each individual for inclusion in our sample reduced our sample size compared to the LEAP sample as a whole. The longitudinal aspect of the study suffered most from this constraint with only 97 participants having a full set of data available at each time point. Additionally, the time between wave 1 and 2 was only an average of 1.5 years in our sample. Given the relatively small longitudinal sample and short time frame for changes to develop, we chose to focus on the main effects of diagnosis and behavioural associations in the current study. The 3rd wave of data collection in LEAP is currently ongoing which has a longer interim period of approximately 8 years. This will provide future researchers the opportunity to delve with greater power into longitudinal trajectories in autism. Our sample size was additionally diminished for certain analyses due to missing clinical data. Most notably the VABS was not available from one site.

Previously it was shown that LICA reproducibility of the first ICs is high, with decreasing reproducibility for subsequent ICs (Llera et al. 2019). Furthermore, reproducibility quickly decreases as the number of participants reduces. Given that our main result is IC62, we expect the reproducibility of this pattern to be low. However, increasing sample sizes may improve reproducibility. Importantly this exploratory analysis directed our attention to the fusiform cortical gradient 2 where we have verified our finding with TSM analysis. Moreover this TSM analysis was reproducible in a split-half analysis (see Additional file 1: subsection 6.1.1). Out of 80 components, only one yielded a significant association with diagnosis when integrating across all modalities. This, combined with the moderate effect size, suggests that group differences are small in the joint measure and do not differ in most brain areas. These findings warrant multimodal integration across larger cohorts to detect more fine-grained differences between the autistic and non-autistic groups.

### Conclusion

Here, we successfully utilised multimodal data integration methods to derive a novel autism-related brain phenotype that revealed group differences in the functional organisation of the right fusiform gyrus. Advanced techniques in unimodal feature extraction enhanced the sensitivity to detect within-ROI functional and structural connectivity changes. Our analysis considered the relative contributions of both structural and functional brain phenotypes simultaneously, uncovering that functional phenotypes seem to drive associations with autism diagnosis and related behavioural measures. Furthermore, these findings expand on previous unimodal approaches implicating the fusiform gyrus in autism by identifying a functional organisation of the fusiform gyrus which relates to autism diagnosis and warrants further investigation. When investigating structural modalities alone we identify a component nominally related to autism diagnosis in line with previous studies [3, 20]. This secondary finding exemplifies the potential of LICA to decompose many structural data domains into ICs that capture significant cross modality covariation.

### Supplementary Information

**Additional file 1.** Supplemental Material.

## Data Availability

The data that support the findings of this study are available from LEAP, the EU-AIMS and AIMS-2-TRIALS programmes, but restrictions apply to the availability of these data, which were used under licence for the current study, and so are not publicly available. Data are, however, available from the authors upon reasonable request and with permission of LEAP and EU-AIMS.

## Abbreviations

ADHD: Attention deficit hyperactivity disorder
ADOS: Autism Diagnostic Observational Schedule
BEDPOSTX: Bayesian Estimation of Diffusion Parameters Obtained using Sampling Techniques
CAT12: Computational anatomy toolbox
CCA: Canonical correlation analysis
DARTEL: Diffeomorphic registration algorithm
dMRI: Diffusion magnetic resonance imaging
DSM-5: Diagnostic and Statistical Manual of Mental, 5th edition
DTI: Diffusion tensor imaging
DWI: Diffusion weighted imaging
ERP: Event related potential
EU-AIMS: European Autism Interventions-A Multicentre Study for Developing New Medications
FD: Framewise displacement
FDR: False-discovery rate
fMRI: Functional magnetic resonance imaging
fsIQ: Full-scale intelligence quotient
FWHM: Full-width half-max
HCP: Human connectome project
IC: Independent component
ICA: Independent component analysis
IQ: Intelligence quotient
LEAP: Longitudinal European Autism Project
LICA: Linked independent component analysis
MCC: Multiple comparison correction
MMI: Multimodality index
MO: Model order
MRI: Magnetic resonance imaging
NT: Neurotypical
PC: Principal component
PCA: Principal component analysis
ProbtrackX: Probabilistic tractography
RBS: Repetitive Behaviour Scale
ROI: Region of interest
RRB: Restricted repetitive behaviour
SD: Standard deviation
SPM: Statistical parametric mapping
SSP: Short sensory profile
SRS: Social Responsiveness Scale
TSM: Trend surface modelling
VABS: Vineland Adaptive Behaviour Scale
VBM: Voxel based morphology
WAIS: Wechsler Abbreviated Scales of Intelligence
WASI: Wechsler Adult Intelligence Scale
WISC: Wechsler Intelligence Scale for Children
