# Supplementary material for: Linking functional and structural brain organisation with behaviour in autism: a multimodal EU-AIMS Longitudinal European Autism Project (LEAP) study
Source: Mol Autism. 2023 Aug 31;14:32. doi: 10.1186/s13229-023-00564-3 (PMC10472578; doi:10.1186/s13229-023-00564-3)
Supplement: Supplementary file 1 — Additional file 1. Supplemental Material. [file 13229_2023_564_MOESM1_ESM.docx]

**SUPPLEMENT
Linking functional and structural brain organisation with behaviour in autism: A multimodal EU-AIMS Longitudinal European Autism Project (LEAP) study**

**Table of Contents**

[**1. Participant demographics 2**](#_heading=h.30j0zll)

[Supplementary Table 1 demographics of individuals at first or only time point 2](#_heading=h.1fob9te)

[Supplementary Table 2 Longitudinal sample demographics at time point 2 2](#_heading=h.3znysh7)

[**2. MRI acquisition parameters 2**](#_heading=h.tyjcwt)

[Supplementary Table 3 Acquisition parameters 3](#_heading=h.3dy6vkm)

[**3. Anatomical preprocessing & QC 4**](#_heading=h.1t3h5sf)

[**4. fMRI preprocessing & QC 5**](#_heading=h.4d34og8)

[**5. DWI preprocessing & QC 6**](#_heading=h.2s8eyo1)

[**6. Results 7**](#_heading=h.17dp8vu)

[6.1 Additional results from function-structure integration 7](#_heading=h.3rdcrjn)

[Supplementary Figure 1 Feature contributions for all 80 components 8](#_heading=h.26in1rg)

[Supplementary Figure 2 Summary of uncorrected results 8](#_heading=h.lnxbz9)

[Supplementary Figure 3 Diagnostic differences in IC62 9](#_heading=h.35nkun2)

[Supplementary Figure 4 Scatterplots of IC3 versus Vineland scales 10](#_heading=h.1ksv4uv)

[Supplementary Figure 5 Spatial maps for IC3 11](#_heading=h.44sinio)

[Supplementary Figure 6 Scatterplots of IC70 versus behavioural scales 12](#_heading=h.2jxsxqh)

[Supplementary Figure 7 Spatial maps for IC70 13](#_heading=h.z337ya)

[6.2 Results from structural only analysis 13](#_heading=h.3j2qqm3)

[Supplementary Figure 8 Summary of uncorrected results of structural only analysis 14](#_heading=h.1y810tw)

[Supplementary Figure 9 Diagnostic differences in IC42 15](#_heading=h.4i7ojhp)

[6.3 Supplementary Figure 10 Spatial maps for IC42 16](#_heading=h.2xcytpi)

[6.3 Results from functional only analysis 16](#_heading=h.1ci93xb)

[Supplementary Figure 11 Summary of uncorrected results from functional only integration 17](#_heading=h.3whwml4)

[Supplementary Figure 12 Diagnostic differences in IC20 18](#_heading=h.2bn6wsx)

[Supplementary Figure 13 Spatial maps of IC20 19](#_heading=h.3as4poj)

[Supplementary Figure 14 Diagnostic differences in IC55 20](#_heading=h.1pxezwc)

[Supplementary Figure 15 Spatial maps for IC55 20](#_heading=h.2p2csry)

[6.4 Comparison with Mei 2020 and Mei 2022 20](#_heading=h.147n2zr)

[Supplementary Figure 16 Comparison of structural IC42 with components from Mei et al (2020, 2022) 21](#_heading=h.3o7alnk)

[**7. References 22**](#_heading=h.23ckvvd)

# Participant demographics

## Supplementary Table 1 demographics of individuals at first or only time point

|  | **Autism** | **Control** | **Test statistic** | **p-value** |
| --- | --- | --- | --- | --- |
| **N** | 206 | 196 |  |  |
| **Sex m:f** | 147:59 | 124:72 | 𝟀^2^= 2.6 | 0.1 |
| **Age mean (SD), years** | 17.8 (5.2) | 17.3 (5.2) | 𝟀^2^ = 393 | 0.5 |
| **IQ mean (SD)** | 101 (20) | 105 (18) | 𝟀^2^ = 188 | 0.05 |
| **Timepoint t1:t2** | 166:40 | 149:47 | 𝟀^2^ = 1 | 0.3 |
| **Site Cambridge**  **KCL**  **Mannheim**  **Nijmegen**  **Utrecht** | 14  74  24  81  13 | 14  67  36  57  22 | 𝟀^2^ = 9 | 0.06 |

## Supplementary Table 2 Longitudinal sample demographics at time point 2

|  | **Autism** | **Control** | **Test statistic** | **p-value** |
| --- | --- | --- | --- | --- |
| **N** | 51 | 46 |  |  |
| **Sex m:f** | 38:13 | 30:16 | 𝟀^2^= 60 | 0.4 |
| **Age mean (SD), years** | 19.2 (5.8) | 17.8 (3.6) | 𝟀^2^ = 96 | 0.5 |
| **IQ mean (SD)** | 101 (18) | 104 (12) | 𝟀^2^ = 73 | 0.3 |
| **Site Mannheim**  **Nijmegen** | 10  41 | 17  29 | 𝟀^2^ = 2.8 | 0.09 |

#

# MRI acquisition parameters

Data used in this study were acquired across 5 sites. Attempts were made to minimise site difference while also optimising acquisition for the different systems available, therefore, slight differences exist in parameters across sites. T1-weighted sequence parameters were based on the ADNI GO/ ADNI 2 protocol. Resting state fMRI data were collected using a multi-echo planar imaging sequence [(Kundu *et al.*, 2012)](https://paperpile.com/c/DbGZMk/69eQ) for a duration of 8-10 minutes. Participants were presented with a cross on the screen and asked to relax, lie still and fixate on the cross for the duration of the scan. Single shell diffusion data were collected with an EPI sequence. See table S3 for full details of all the scan parameters.

## Supplementary Table 3 Acquisition parameters

| **Scanner Hardware** | | | | | |
| --- | --- | --- | --- | --- | --- |
| **Site** | Cambridge | London | Mannheim | Nijmegen | Utrecht |
| **Manufacturer, type** | Siemens, Prisma Fit | GE,  MR750 | Siemens, TIM Trio | Siemens, Skyra | Philips,  Ingenia CX |
| **Field strength** | 3T | 3T | 3T | 3T | 3T |
| **Headcoil** | 12-ch | 8-ch | 12-ch | 32-ch | 8-ch |
| **MPRAGE T1-weighted sequence** | | | | | |
| **TR / TE (ms)** | 2300/2.95 | 7310/3.02 | 2300/2.93 | 2300/2.93 | 6760/3.1 |
| **Flip angle (°)** | 9 | 11 | 9 | 9 | 9 |
| **In-plane resolution (mm^2^)** | 1.1 x 1.1 | 1.1 x 1.1 | 1.1 x 1.1 | 1.1 x 1.1 | 1.1 x 1.1 |
| **Slice thickness (mm) / No.** | 1.2 / 176 | 1.2 / 196 | 1.2 / 176 | 1.2 / 176 | 1.2 / 170 |
| **EPI BOLD resting state sequence** | | | | | |
| **TR / TE_1_ / TE_2_ / TE_3_ (ms)** | 2300 /12/29/46 | 2300/12.7/31/ 48 | 2300/12/29/46 | 2300/12/29/46 | 2300/13/31/49 |
| **Flip angle (°)** | 80 | 90 | 80 | 80 | 80 |
| **Slice thickness (mm) / No.** | 3.8 / 33 | 3.8 / 33 | 3.8 / 33 | 3.8 / 33 | 3.75 / 33 |
| **In-plane resolution (mm^2^)** | 3.8 x 3.8 | 3.8 x 3.8 | 3.8 x 3.8 | 3.8 x 3.8 | 3.75 x 3.75 |
| **No. of measurements** | 266 | 215 | 215 | 266 | 200 |
| **EPI diffusion weighted sequence** | | | | | |
| **TR / TE (ms)** | 1200 / 67 | 1200 / 70 | 1200 / 103 | 1200 / 102 | 1200 / 96 |
| **Flip angle (°)** | 90 | 90 | 90 | 90 | 90 |
| **Slice thickness (mm) / No.** | 2 / 72 | 2 / 72 | 2 / 72 | 2 / 72 | 2 / 72 |
| **In-plane resolution (mm^2^)** | 2 x 2 | 2 x 2 | 2 x 2 | 2 x 2 | 2 x 2 |
| **B-values (s/mm^2^)** | 0 / 1500 | 0 / 1500 | 0 / 1500 | 0 / 1500 | 0 / 1500 |
| **No. of gradients** | 6 / 60 | 6 / 60 | 6 / 60 | 6 / 60 | 6 / 60 |

Abbreviations: Mannheim - Central Institute of Mental Health, Mannheim, Germany; London - Institute of Psychiatry, Psychology and Neuroscience, King’s College London, United Kingdom; Nijmegen - Radboud University Nijmegen Medical Centre, the Netherlands; Cambridge - Autism Research Centre at the University of Cambridge, United Kingdom; Utrecht - University Medical Centre Utrecht, the Netherlands. GE - General Electric (General Electric Medical Systems, Milwaukee, WI, USA); Philips (Philips Healthcare Systems, Best, The Netherlands); Siemens (Siemens, Erlangen, Germany). ch - channel; TE - echo time; TI - inversion time; TR - repetition time.

# Anatomical preprocessing & QC

All available raw T1w-MRI scans from LEAP time point 1 and time point 2 participants were visually assessed for quality. We looked for duplicate scans or other marked brain anomalies, such as unusually large ventricles. The scans that passed this first quality control went on for further QC. Subjects were excluded due to significant artefacts in the scans or brain anomalies which made the normalisation to MNI standard space problematic.

After the initial quality control, the remaining images were processed using the manual of the computational anatomy toolbox (CAT12: <http://www.neuro.uni-jena.de/cat/>) in statistical parametric mapping software (SPM12; Wellcome Department of Imaging Neuroscience, London, UK). Firstly, the toolbox was utilised to segment the tissues within the individual anatomical MRI scans. Specifically, the tissues within the scans are classified based on intensity values, which allows an algorithm to segment the scans into grey matter, white matter, and cerebrospinal fluid [(Ashburner and Friston, 1997)](https://paperpile.com/c/DbGZMk/yM2U). This is basically achieved by registering inconsistencies in the magnetic field picked up by the MRI scanner and inferring inconsistencies in the underlying tissues from this [(Kurth, Luders and Gaser, 2015)](https://paperpile.com/c/DbGZMk/23gr). To guide this process, we supplied the algorithm with tissue probability maps of where specific tissues are to be expected. This process generated one grey matter, one white matter, and one cerebrospinal fluid volume estimation map per scan. Here, some subjects were excluded due to tissue segmentation issues reported by QC reports issued by the CAT12 SPM12 pipeline. Then, to make the segmented grey matter maps comparable across subjects, the scans were spatially normalised and smoothed in subsequent steps.

Consequently, the grey matter maps were then used to generate a study-specific template using high-dimensional, nonlinear DARTEL templates [(Ashburner, 2007)](https://paperpile.com/c/DbGZMk/b0uT). Such a template is used to achieve more accurate inter-subject registration of brain images and more accurate tissue segmentation. Like in other unimodal studies we preserved the information within each voxel on local tissue volume by including a Jacobian modulation step using flow fields (Mei et al., 2020). As our data was partially longitudinal, we limited the template generation to only one scan per individual so as not to bias the template. The template was then normalised to MNI152 standard space.

Once the template was generated, the anatomical scans of all subjects were projected onto the templates and normalised in the process since the templates themselves were already in MNI152 space. After registration and normalisation, the scans were spatially smoothed using a full-width half-max (FWHM) isotropic Gaussian kernel of 4 mm. Smoothing was done for several reasons, including but not limited to the further reduction of small differences between grey matter density maps (Kurth, Luders & Gaser, 2015). After smoothing, the grey matter density maps were ready to be used in statistical analyses to determine grey matter density abnormalities. Since we implemented an integrative analysis using LICA, all the grey matter density maps were concatenated into one 4D (dimensional) file where the 4th dimension were the subjects. To reduce the computational load experienced during LICA on the cluster we downsampled the merged VBM before integration to 2mm isotropic voxel dimension.

# fMRI preprocessing & QC

After recombining the three rs-fMRI scan echoes using echo-time weighted averaging, the data were preprocessed using a standard preprocessing pipeline that included tools from the FMRIB Software Library (FSL version 5.0.6; http://www.fmrib.ox.ac.uk/fsl). Preprocessing included removal of the first five volumes to allow for signal equilibration, primary head motion correction via realignment to the middle volume (MCFLIRT; [(Jenkinson *et al.*, 2002)](https://paperpile.com/c/DbGZMk/VZ7d)) and grand mean scaling. Next, we thoroughly corrected for secondary head motion related artefacts, by applying ICA-AROMA, an ICA-based method, which automatically detects and removes motion-related components from the data [(Pruim *et al.*, 2015)](https://paperpile.com/c/DbGZMk/Rk1w). Following this, we applied nuisance regression to remove signal from white matter and cerebrospinal fluid and a high-pass filter (0.01 Hz).

The rs-fMRI images of each participant were coregistered to the participants' anatomical images via boundary-based registration implemented in FSL FLIRT [(Greve and Fischl, 2009)](https://paperpile.com/c/DbGZMk/N7wh). The T1 images of each participant were registered to MNI152 standard space using an affine transformation with 12 degrees of freedom and refined using non-linear registration with FSL FNIRT (10mm warp, 2mm resampling resolution; [(Jenkinson *et al.*, 2002)](https://paperpile.com/c/DbGZMk/VZ7d)). Then, we brought all participant-level rs-fMRI images to 2mm MNI152 standard space by applying the R-fMRI to T1 and T1 to MNI152 transformations. Finally, we smoothed the data with a Gaussian kernel of sigma 2.5 mm, corresponding to a FWHM of approx = 5.9mm.

# DWI preprocessing & QC

First, de-noising was performed using the MP-PCA approach [(Veraart, Fieremans and Novikov, 2016)](https://paperpile.com/c/DbGZMk/SQVG). This method identifies noise in the MRI data and removes it using a principal component analysis by applying a random matrix theory approach. Second, Gibbs-ringing artefacts were removed following methods described in [(Kellner *et al.*, 2016)](https://paperpile.com/c/DbGZMk/jbBL). Eddy-current induced distortions and subject motion were then corrected using the FSL tool “eddy”, which uses a model-free estimation of all distortions while it also detects and replaces slice outliers in the DWI data [(Andersson and Sotiropoulos, 2016)](https://paperpile.com/c/DbGZMk/iqln). Intra-volume slice motion correction was introduced to improve the final quality of data and recover most of the data with motion artefacts [(Andersson *et al.*, 2017)](https://paperpile.com/c/DbGZMk/AWmL). Quality control reports were then generated for each subject and each site [(Bastiani *et al.*, 2019)](https://paperpile.com/c/DbGZMk/sbja). Few subjects with excessive motion during acquisition (absolute motion > 4 mm translation), with high number of outliers (>6%) were excluded from the study. To also correct for possible signal drop-out in the b0 data, b0 voxels for each slice were compared and outliers were identified using the interquartile range interval approach for outlier detection (threshold = Q3 + 1.5 IQR). Slices that were considered outliers were then replaced with the corresponding median b0. Finally, to make the diffusion data more uniform across sites, all data were resampled using a real symmetric spherical harmonics representation limited to l=6. This allowed a further reduction of high frequency noise and allowed the detection and replacement of remaining data outliers not fully recovered by eddy. Because some of the datasets from Siemens scanners still exhibited residual Gibbs ringing in b0s data compared to the other sites, a final Gibbs ringing correction was applied to all datasets using ExploreDTI only to b0 data [(Perrone *et al.*, 2015)](https://paperpile.com/c/DbGZMk/hwdr). After visual inspection data quality looked consistent across datasets and with no visible residual artefacts.

Please note, because Nijmegen and Mannheim exhibited a high Rician noise floor in the raw data [(Sotiropoulos *et al.*, 2013)](https://paperpile.com/c/DbGZMk/58Fy), before running the main preprocessing pipeline, datasets from these sites were pre-corrected using an in-house implementation of the methods described in [(Koay and Basser, 2006)](https://paperpile.com/c/DbGZMk/GGcR) to reduce the bias in the DWI signal induced by the noise floor.

# Results

## 6.1 Additional results from function-structure integration


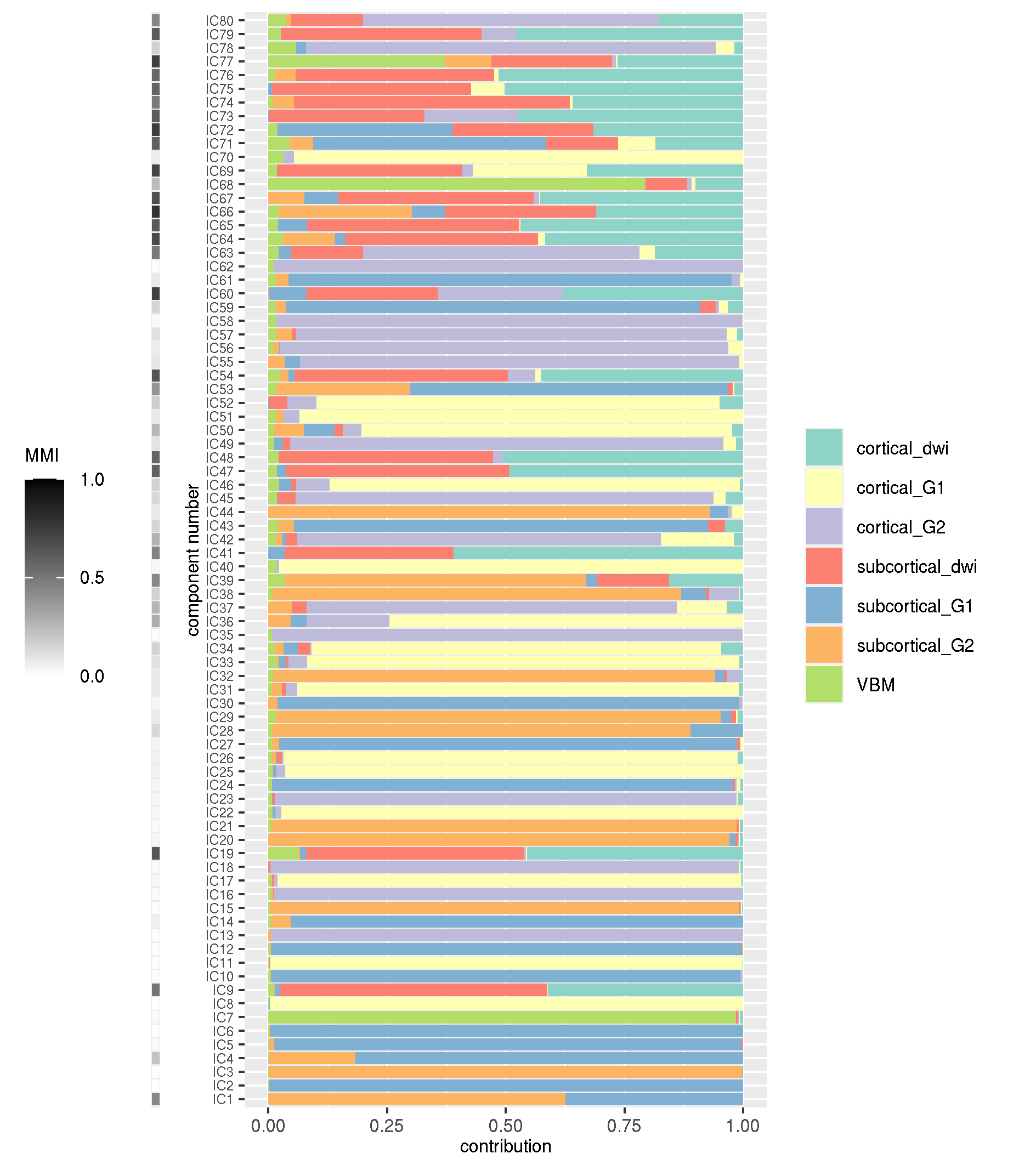


## Supplementary Figure 1 Feature contributions for all 80 components

Modality contributions for all generated components are shown. On the left-hand side the MMI - multimodal index - is shown, which indicates if a component is driven by one modality (MMI = 0) or equally by all modalities (MMI = 1). IC - independent component, VBM - Voxel Based Morphometry, dwi - diffusion weighted imaging, G1/2 - gradient 1/2.


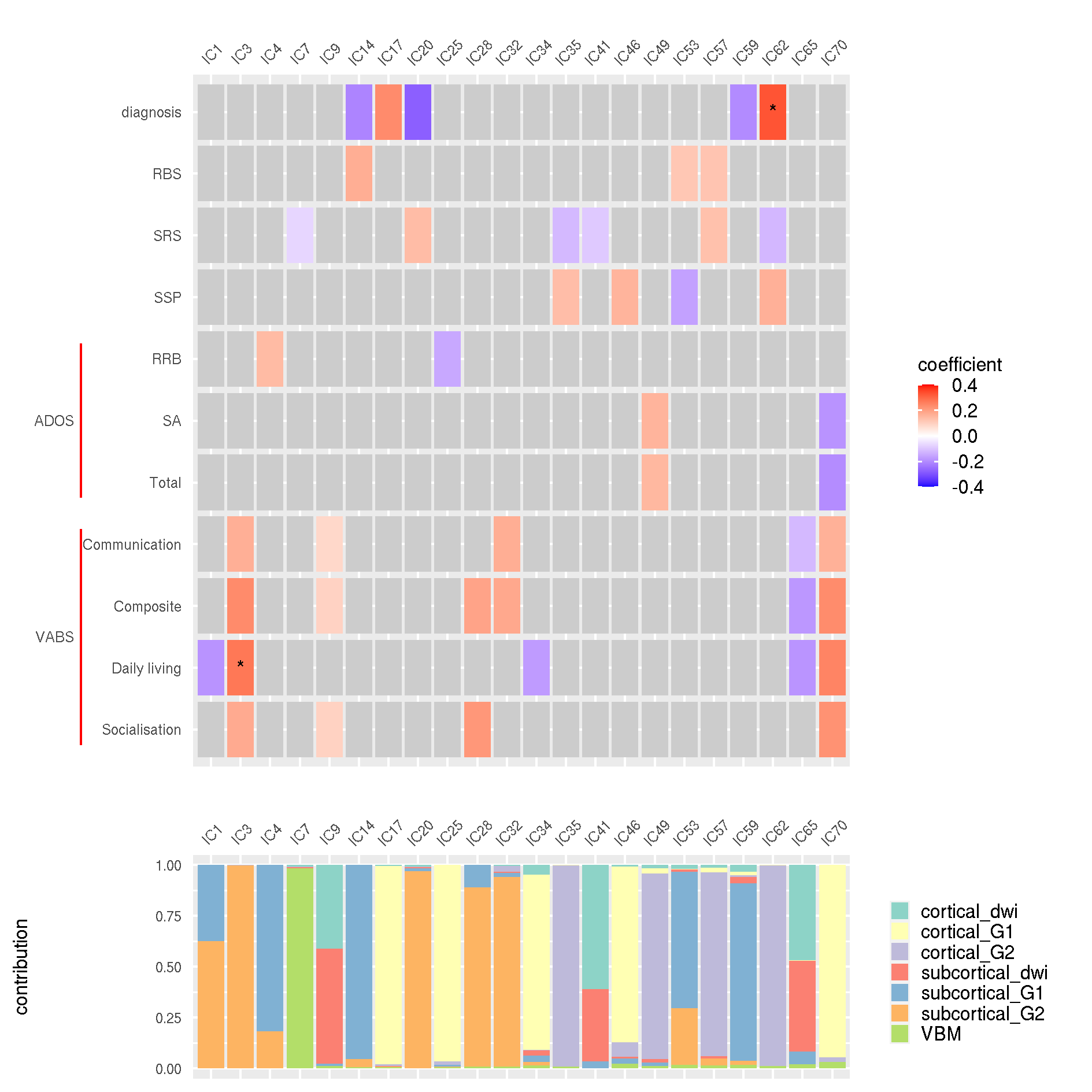


## Supplementary Figure 2 Summary of uncorrected results

The top figure is a heatmap of significant uncorrected results for all components with findings. All coloured tiles were nominally significant. * *p_uncorrected_* < 0.001. The bottom figure shows the corresponding feature contributions for these components. ADOS - Autism Diagnostic Observational Schedule, VABS - Vineland Adaptive Behaviours Scale, RBS - Repetitive Behaviours Scale, SRS - Social Responsiveness Scale, SSP - Short Sensory Profile, RRB - Restrictive and Repetitive Behaviours scale, SA - Social Affect scale, IC - independent component, VBM - Voxel Based Morphometry, dwi - diffusion weighted imaging, G1/2 - gradient 1/2.


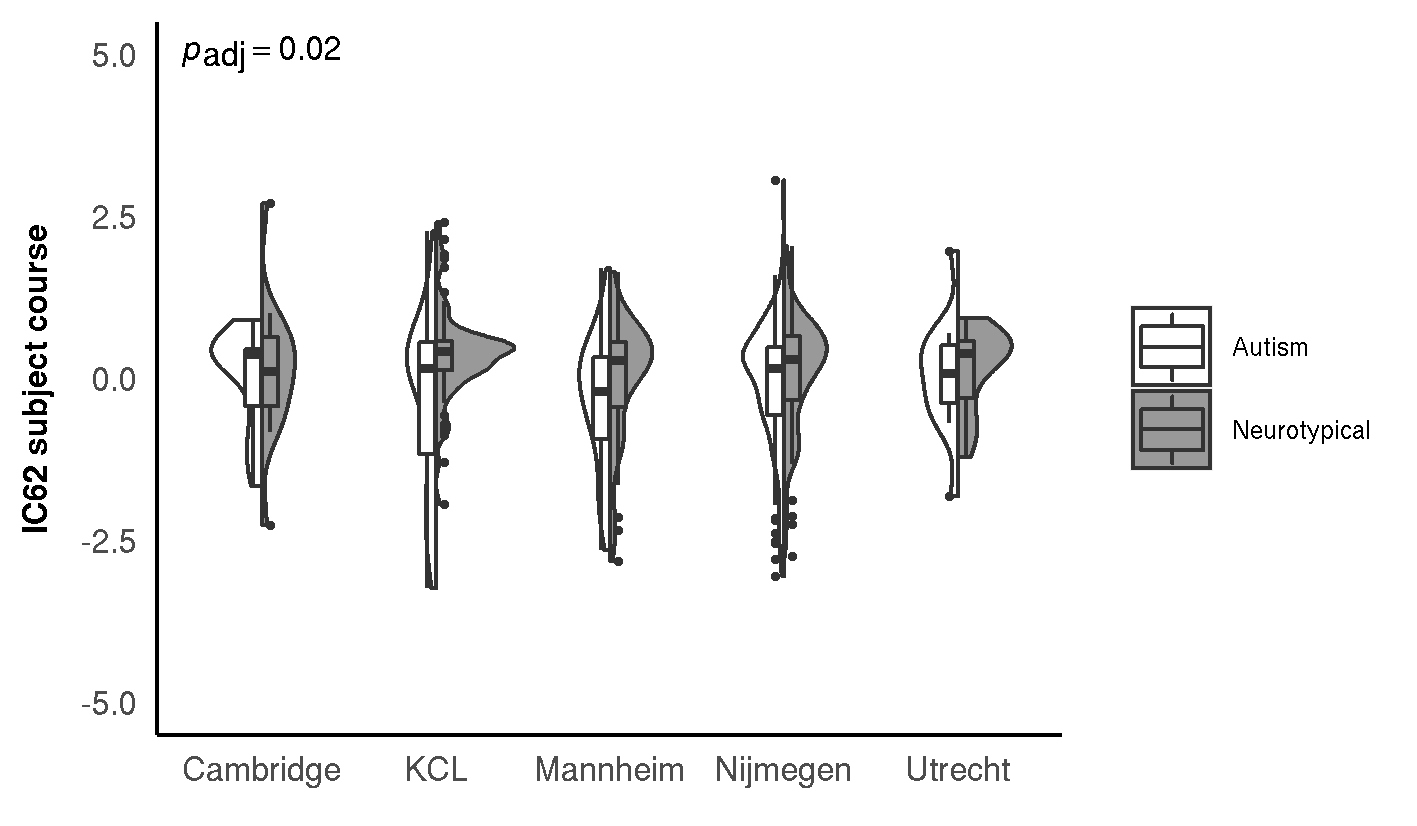


## Supplementary Figure 3 Diagnostic differences in IC62

Violin-plot with nested box-plot showing the IC62 subject course against the diagnostic groups across all involved acquisition sites. The violin plots show the distribution of the subject courses while the boxplots indicate the first and third quartile with the median denoted with a thick horizontal line. The unadjusted and FDR-adjusted p-values are shown in the top left corner. There was a significant main effect of group. We found no significant site effect or diagnosis-by-site interaction effect.

IC62 survives multiple comparison correction (MCC) for the number of components tested and is reported in the main text. In the analysis of diagnosis no significant association was found with other components that survive MCC (*p_adj_ > 0.05)*. Additionally, no significant associations with behavioural measures were found after correction for multiple comparisons. The significant effect of group was not influenced by site, which is shown split into individual sites above (Figure S3). However, there were multiple components (N=22) that were nominally associated with multiple behavioural measures and/or the autism diagnosis. These are summarised in the above heatmap (Figure S2). Below we additionally provide further information on the nominal results from IC3 and IC70. These were selected due to their low p value and nominal association with multiple variables, respectively.

We find nominally significant associations between IC3 and multiple Vineland measures (Figure S4) (Vineland composite: coeff.=0.24, *p_unadj_*=0.004; Vineland socialisation: coeff.=0.18, *p_unadj_*=0.004; Vineland daily living: coeff.=0.27, *p_unadj_*=0.0008; Vineland communication: coeff.=0.16, *p_unadj_*=0.03). In the spatial maps we observe that the component is mainly driven by functional subcortical gradient 2 (99.68%) in the bilateral putamen, caudate, and the amygdala (Figure S5). Of note, the spatial maps are shown at |z|>0.7 because other thresholds were too high, thus these spatial features should be interpreted with caution. The amygdala and the caudate show a weak negative effect bilaterally, while the putamen shows a weak negative effect in the right hemisphere, and a weak positive effect in the left hemisphere.


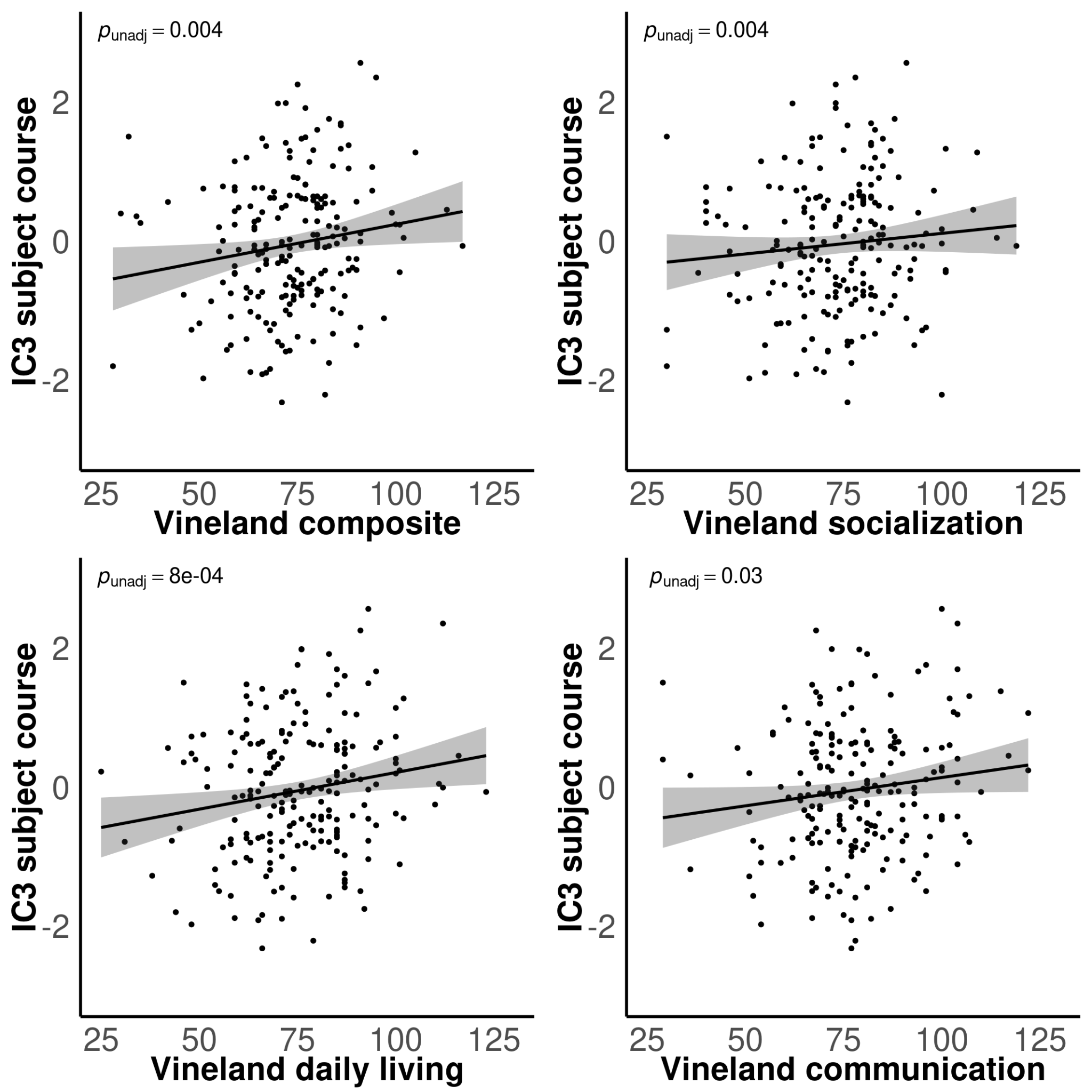


## Supplementary Figure 4 Scatterplots of IC3 versus Vineland scales

The Vineland daily living, composite and communication scores are plotted against the IC3 subject course. The black line represents the line of best fit, with the grey area around it showing the standard error. The FDR unadjusted p-value is shown in the top-left corner of each plot. We find a slightly positive correlation across all metrics. None of these associations survive MCC.

## Supplementary Figure 5 Spatial maps for IC3
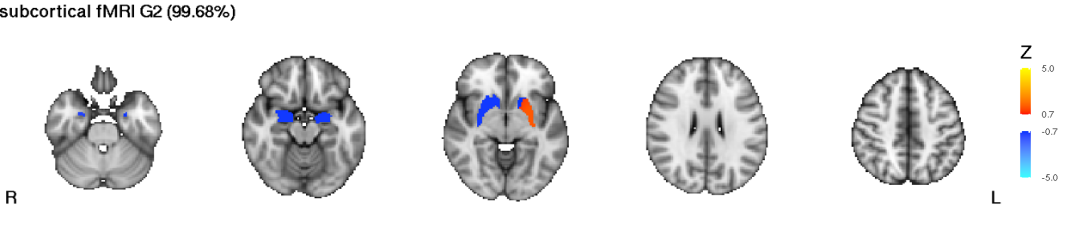


Modalities contributing <1% are excluded from visualisation. The scale represents the Z-score of spatial contribution within each feature. |z| > 0.7 is shown.

Component IC70 shows nominal association with all Vineland measures and some ADOS measures (Figure S6). Specifically, we find a negative relationship between the subject contributions to IC70 and the total ADOS score (coeff.=-0.20, *p_unadj_*=0.004), and the ADOS social affect scale (coeff.=-0.19, *p_unadj_*=0.007). Furthermore, we find a slight positive correlation with all Vineland measures (Vineland composite: coeff.=0.24, *p_unadj_*=0.001; Vineland socialisation: coeff.=0.22, *p_unadj_*=0.001; Vineland daily living: coeff.=0.25, *p_unadj_*=0.001; Vineland communication: coeff.=0.16, *p_unadj_*=0.005). The spatial maps show that the component is mainly driven by the cortical connectopic gradient 1 (94.66%) with both the bilateral fusiform gyrus and postcentral gyrus showing positive contributions (Figure S7). Additionally, the VBM contributed 3.2% to the component with dispersed contributions of grey matter density, and the cortical gradient 2 contributed 2.12%.


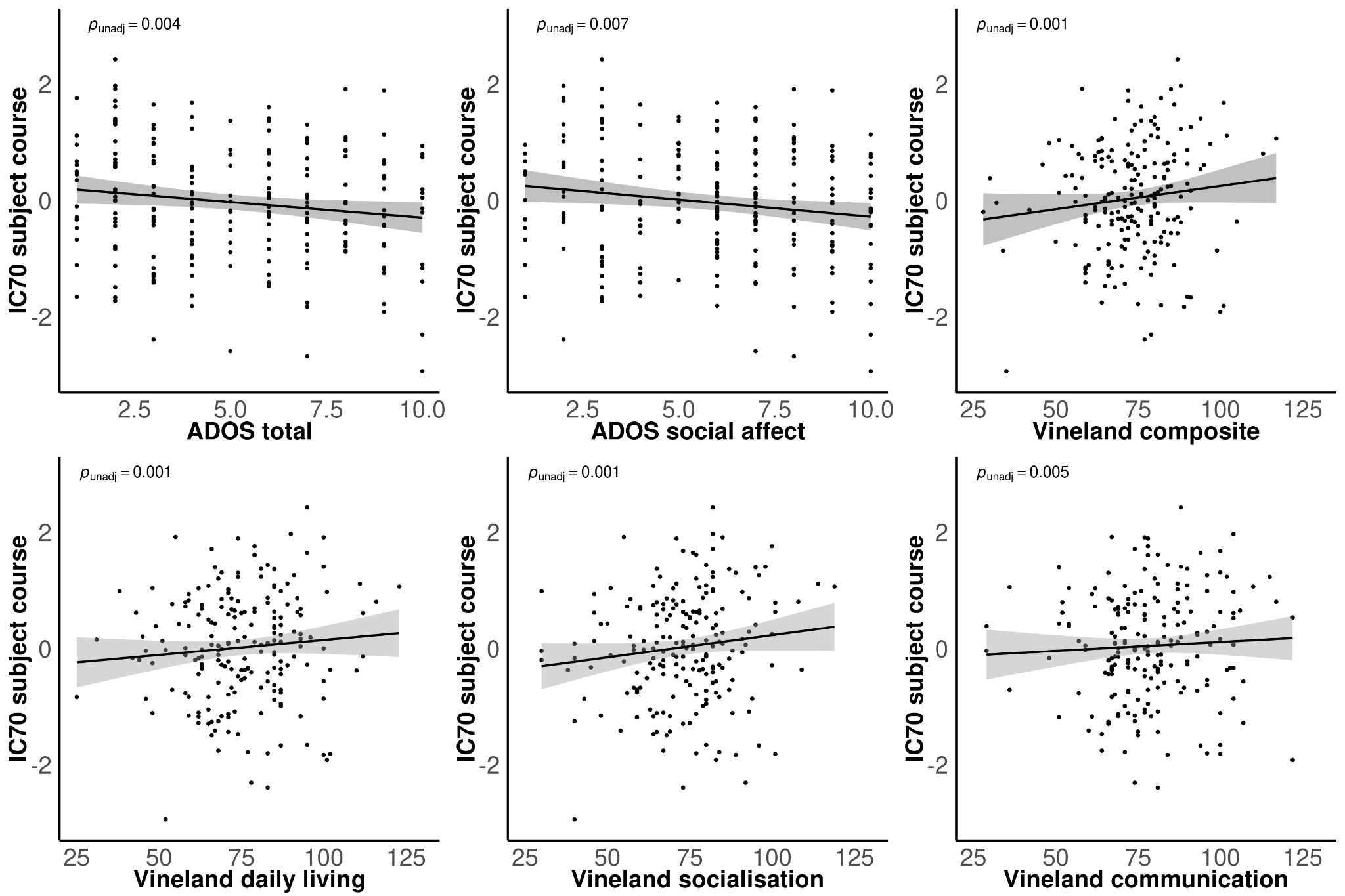


## Supplementary Figure 6 Scatterplots of IC70 versus behavioural scales

The ADOS total score, ADOS social affect score, Vineland daily living, composite and communication scores are plotted against the IC70 subject course. The black line within each plot represents the line of best fit, with the grey area around it showing the standard error. The unadjusted p-value for each correlation is shown in the top-left corner of each plot respectively. We find a slightly positive association across all Vineland metrics, and a slightly negative association across ADOS measures. None of these associations survive MCC. ADOS - Autism Diagnostic Observational Schedule.


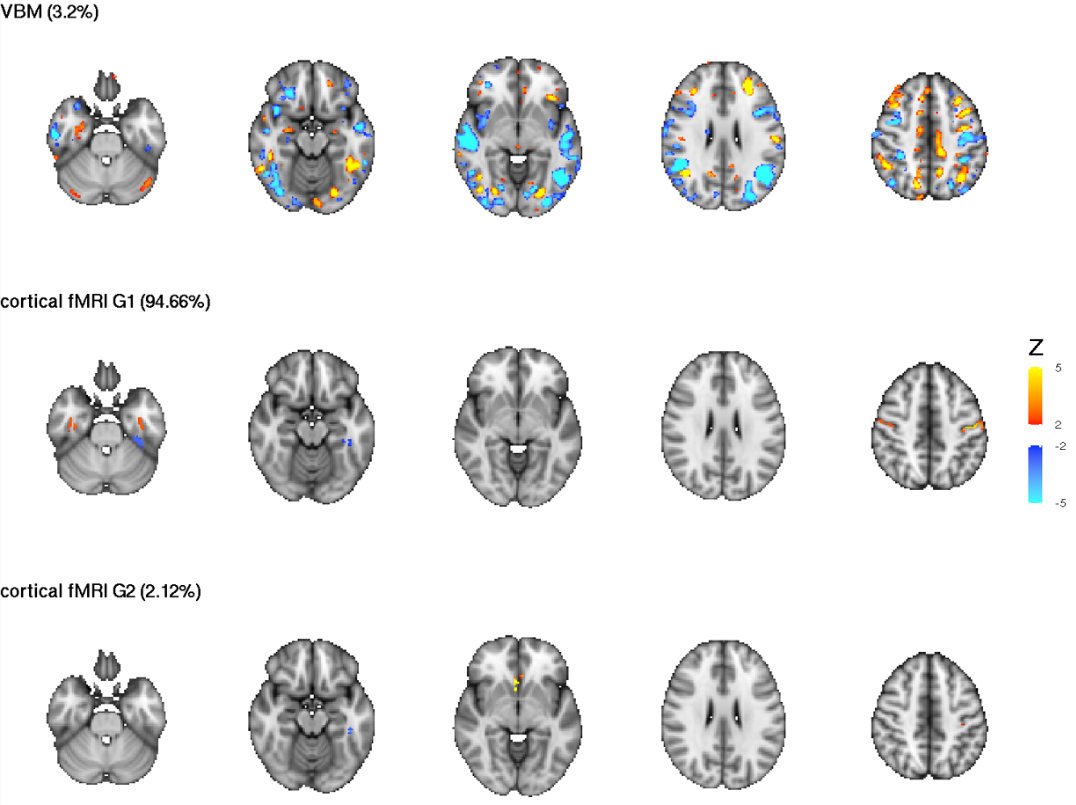


## Supplementary Figure 7 Spatial maps for IC70

Modalities contributing <1% are excluded from visualisation. The scale represents the Z-score of spatial contribution within each feature. |z| > 2 is shown.

## 6.1.1 TSM split-half analysis

To determine the reproducibility of the TSM analysis we randomly split the data into almost equally sized halves (split 1 = 250 scans, split 2 = 249 scans). Then, we calculated the associations using a GLME-model with the same parameters as in the main analysis. Across both halves a similar pattern of effect was seen in the three TSM coefficients (1, 6, 7) that were significant in the full analysis. We found that TSM coefficient 6 was significantly associated with group in one half (split 1: coefficient=0.07, padj=0.022) and trend level significant in the second half (split 2: coefficient=0.07, padj=0.063). Similarly TSM coefficient 7 was significantly associated with group in one half (split 2: coefficient=0.03, padj=0.046) and trend level in the other (split 1: coefficient=0.03, padj=0.083). Finally coefficient 1 was significantly associated with group in the second half (split 2: coefficient=-0.13, padj=0.043) but not the first (split 1: coefficient=-0.09, padj=0.5) although the direction of effect was consistent.

## 6.1.2 Post-hoc analysis accounting for non-independence

To address calls for a follow-up analysis that accounts for the non-independence between scans we constrained the included scans to one per subject. Thus, we performed the LICA decomposition with N=402 individuals and repeated the statistical analysis using a generalised linear model instead of mixed-effects model. The analysis showed multiple components related to diagnosis prior to multiple comparison correction. IC71 had the lowest p-value (0.0025) of these (figure S8). However, it did not survive multiple comparison correction when accounting for all 80 components tested. IC71 strongly reflected IC62 from the initial analysis. This component was similarly strongly driven by cortical gradient G2 (92%) and strongly influenced by the right fusiform gyrus (figure S9). The correlation between the cortical G2 maps from IC62 and IC72 was r=0.37. Additionally, the correlation between subject courses from the overlapping subjects in these ICs revealed high correspondence (rho = -0.47, p<0.001).


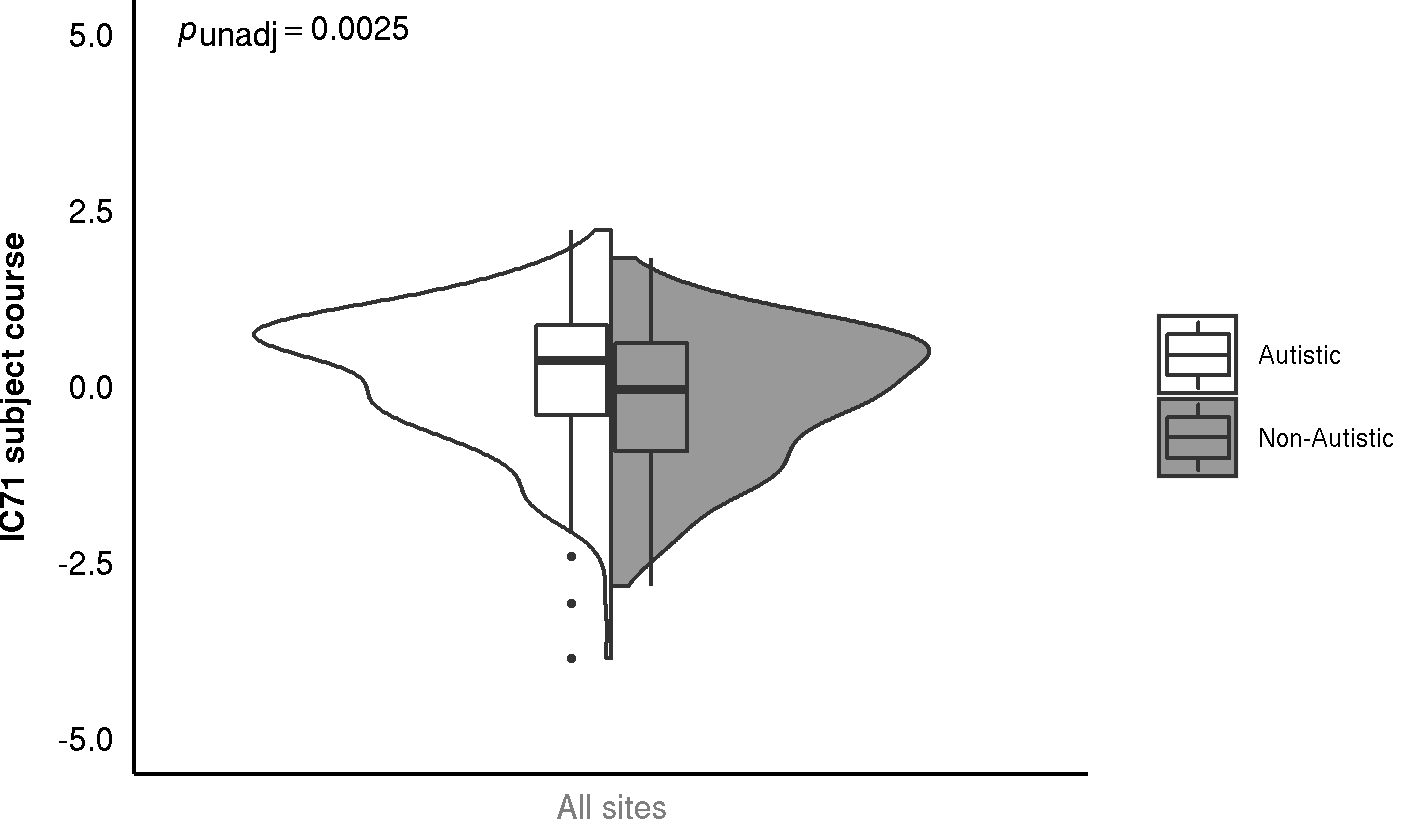


## Supplementary Figure 8 IC71 subject course per group

The violin plot shows the distribution of the subject courses while the boxplots indicate the first and third quartile with the median denoted with a thick horizontal line. There was a nominally significant main effect of diagnostic group. The unadjusted p-value is shown in the top left corner.


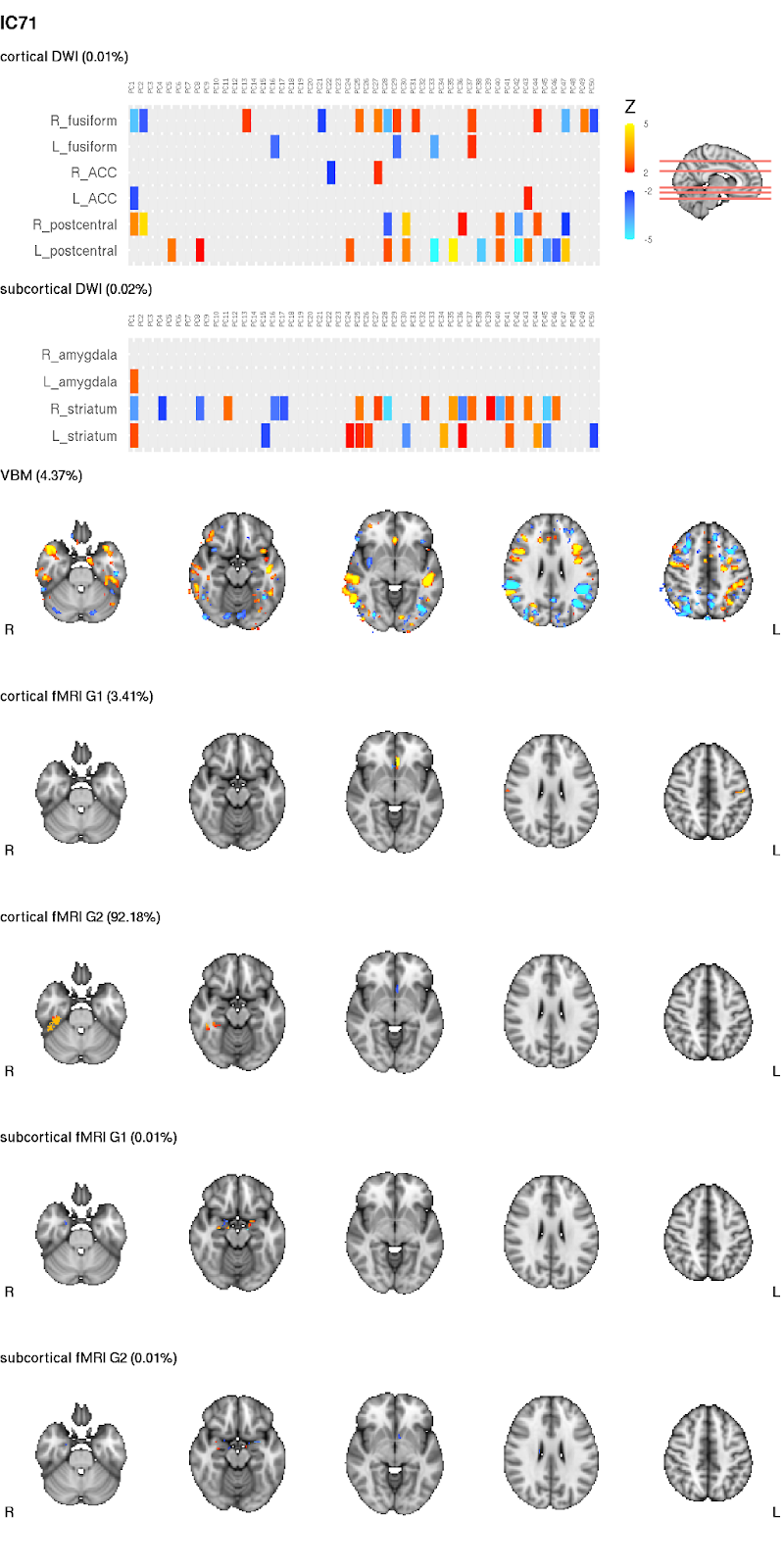


## Supplementary Figure 9 Spatial maps for IC71

Modalities contributing <3% are excluded from visualisation. The scale represents the Z-score of spatial contribution within each feature. |z| > 2 is shown.

## 6.2 Results from structural only analysis

When decomposing our data for only structural modalities, namely DWI and VBM brain metrics, we found N=30 ICs nominally associated with the autism diagnosis and/or with the behavioural/clinical measures investigated (Figure S8).


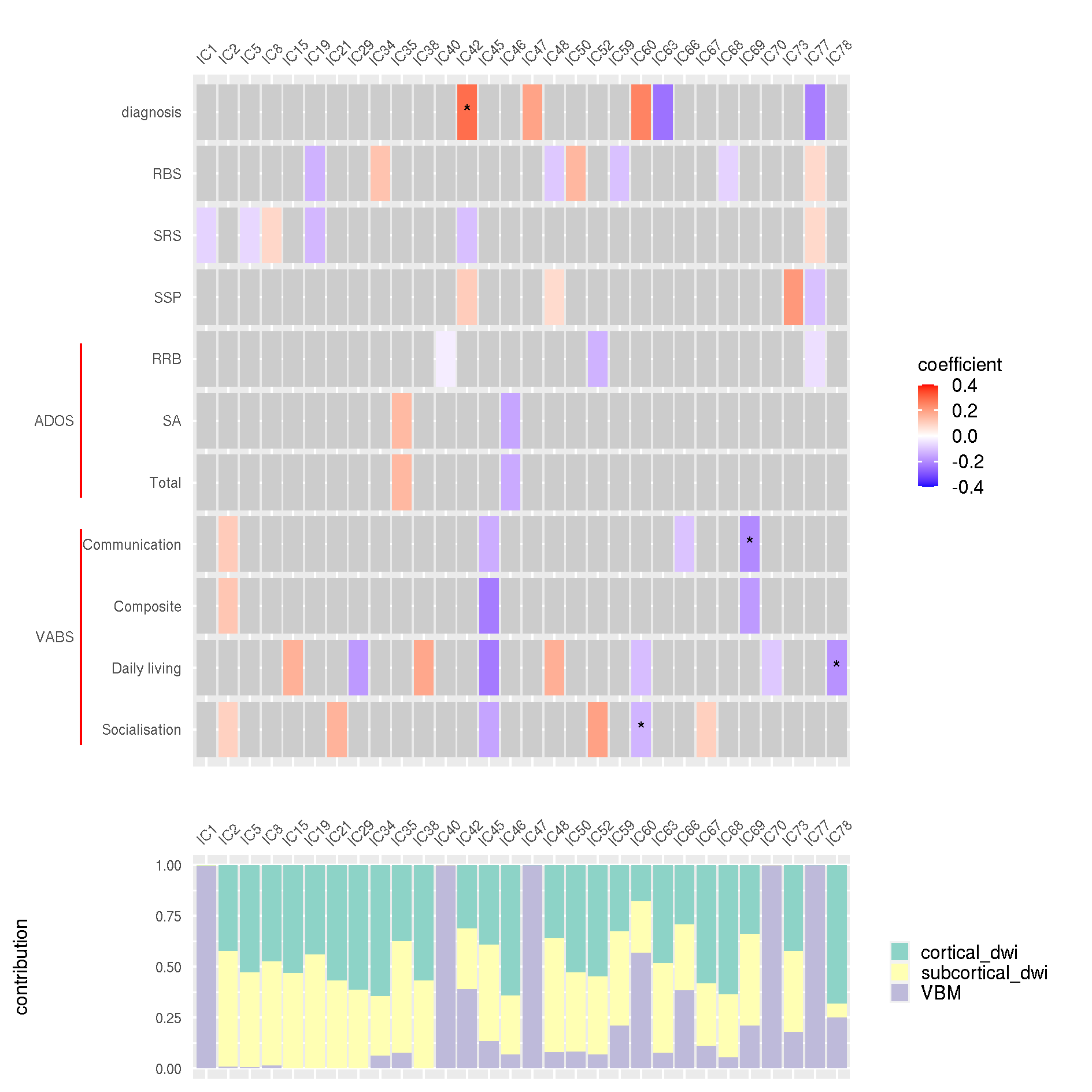


## Supplementary Figure 10 Summary of uncorrected results of structural only analysis

The top figure is a heatmap of significant uncorrected results for all components with findings. All coloured tiles were nominally significant. * *p_uncorrected_* < 0.001. The bottom figure shows the corresponding feature contributions for these components. ADOS - Autism Diagnostic Observational Schedule, VABS - Vineland Adaptive Behaviours Scale, RBS - Repetitive Behaviours Scale, SRS - Social Responsiveness Scale, SSP - Short Sensory Profile, RRB - Restrictive and Repetitive Behaviours scale, SA - Social Affect scale, IC - independent component, VBM - Voxel Based Morphometry, dwi - diffusion weighted imaging.

One component, IC42, was significantly related to autism diagnosis before MCC; this association did not survive MCC when considering all components tested (coeff.=0.29, *p_adj_*=0.054, Figure S9). On this association we found significant site (χ^2^=158.1, *p*=2*10^-16^), sex (χ^2^=10.6, *p*=0.001), age (χ^2^=5.3, *p*=0.02) and site-by-diagnosis (χ^2^=10.4, *p*=0.03) effects. Additionally, the nominal diagnosis effect was not robust to the inclusion of a site-by-diagnosis interaction term in the model (χ^2^=0.9, *p*=0.34) revealing this finding was not robust or generalisable. Nevertheless, VBM, cortical and subcortical DWI features all contributed to this component, with the cortical DWI contributing 31.18%, the subcortical DWI contributing 29.96% and VBM contributing 38.86% (Figure S10).

Additional analyses of continuous brain-behaviour relationships revealed multiple nominal associations (Figure S8) but no significant relationships after MCC (*p*>0.05).


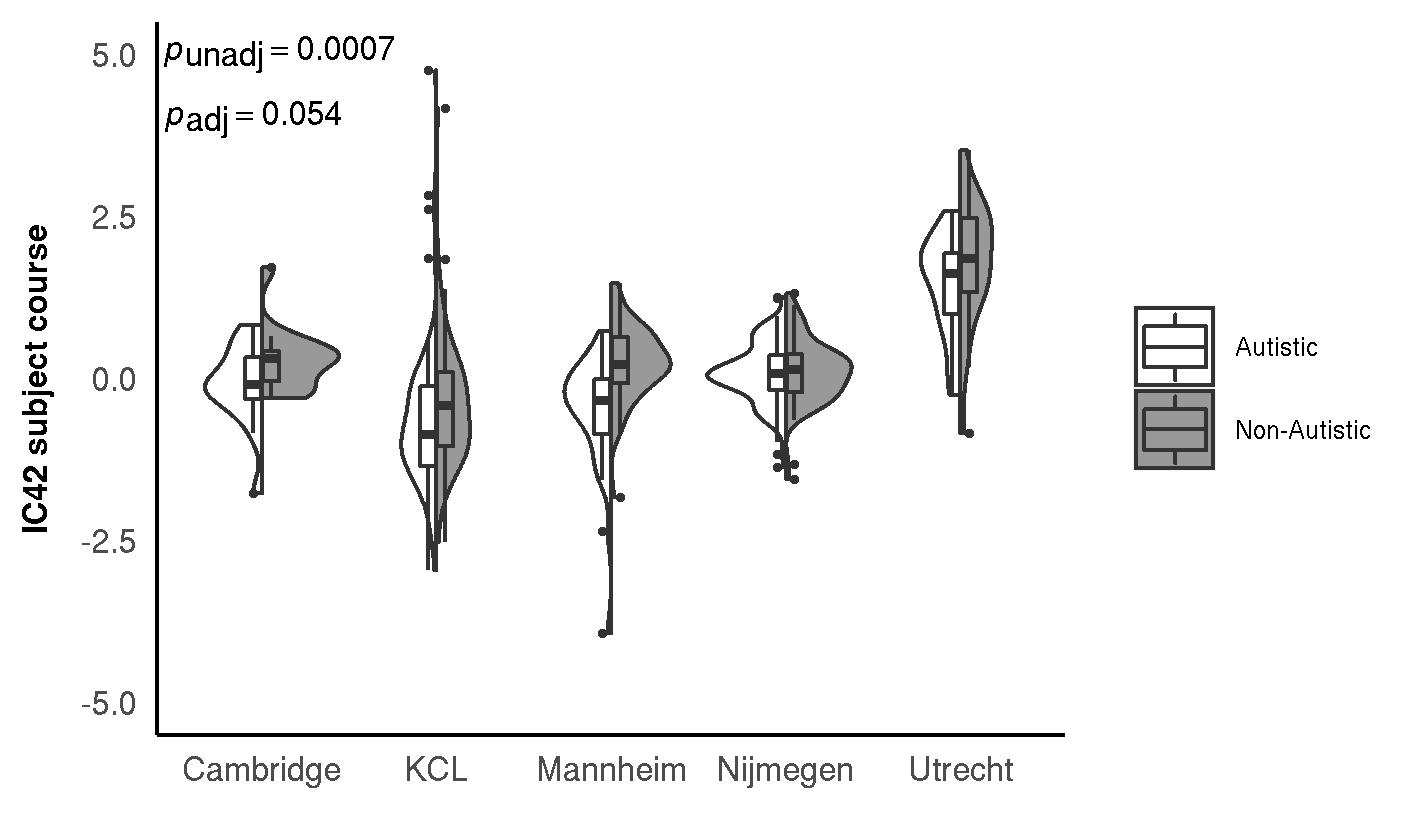


## Supplementary Figure 11 Diagnostic differences in IC42

Violin-plot with nested box-plot showing the IC42 subject course against the diagnostic groups across all involved acquisition sites. The violin plots show the distribution of the subject courses while the boxplots indicate the first and third quartile with the median denoted with a thick horizontal line. The unadjusted and FDR-adjusted p-values are shown in the top left corner. There was a significant main effect of group and acquisition site. Furthermore, there was a significant diagnosis-by-site interaction.

##
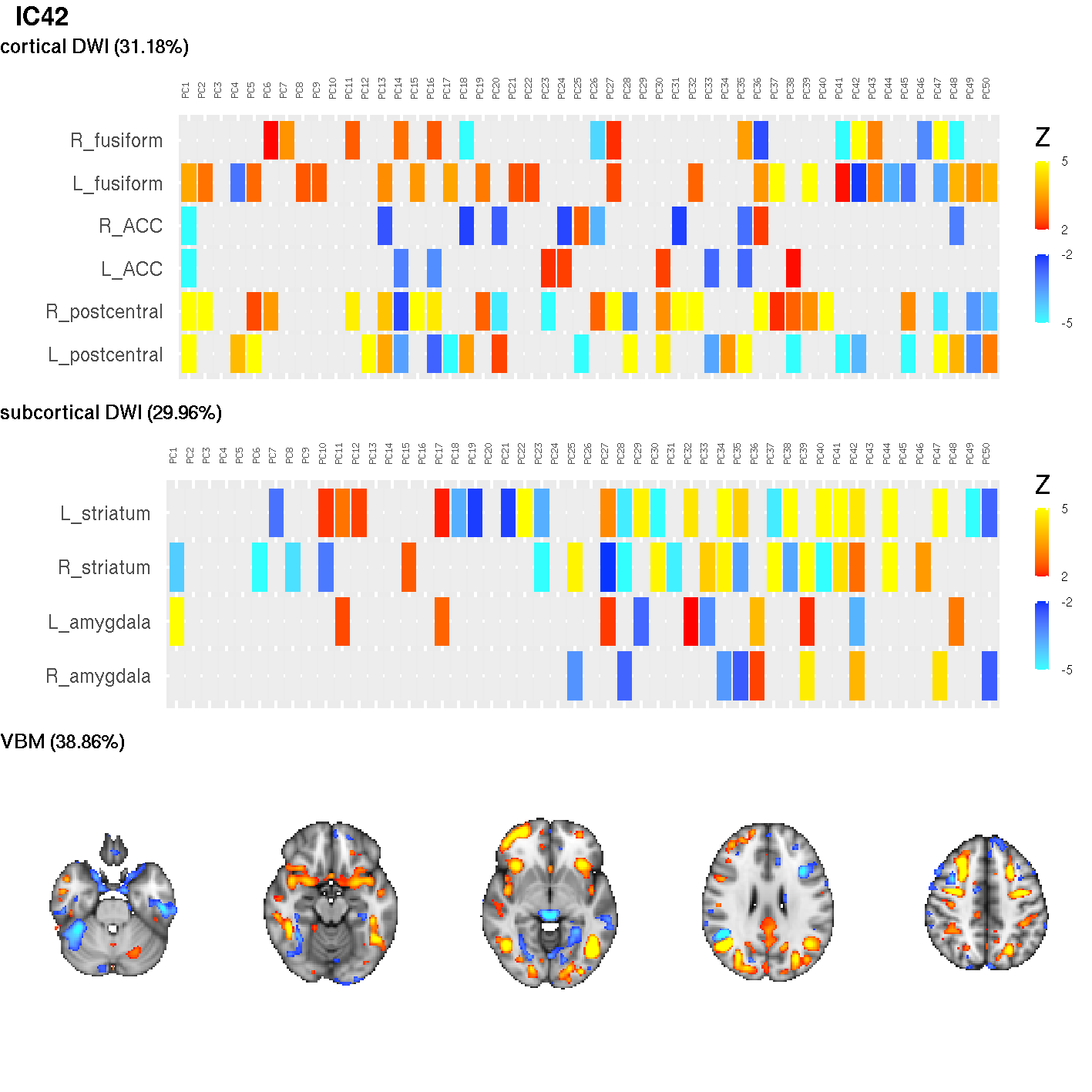
6.3 Supplementary Figure 12 Spatial maps for IC42

Modality contributions of IC42 are shown. The scale represents the Z-score of spatial contribution within each feature. PC’s 1-50 of the DWI data are visualised. |z| > 2 is shown.

## 6.3 Results from functional only analysis

When decomposing our data for only functional modalities, namely connectopic gradients derived from rs-fMRI, we found N=26 components nominally associated with either autism diagnosis or behavioural/clinical metrics tested in the present paper (Figure S11). Of the components, two were significantly associated with autism diagnosis after MCC for the number of components tested, IC20 and IC55.


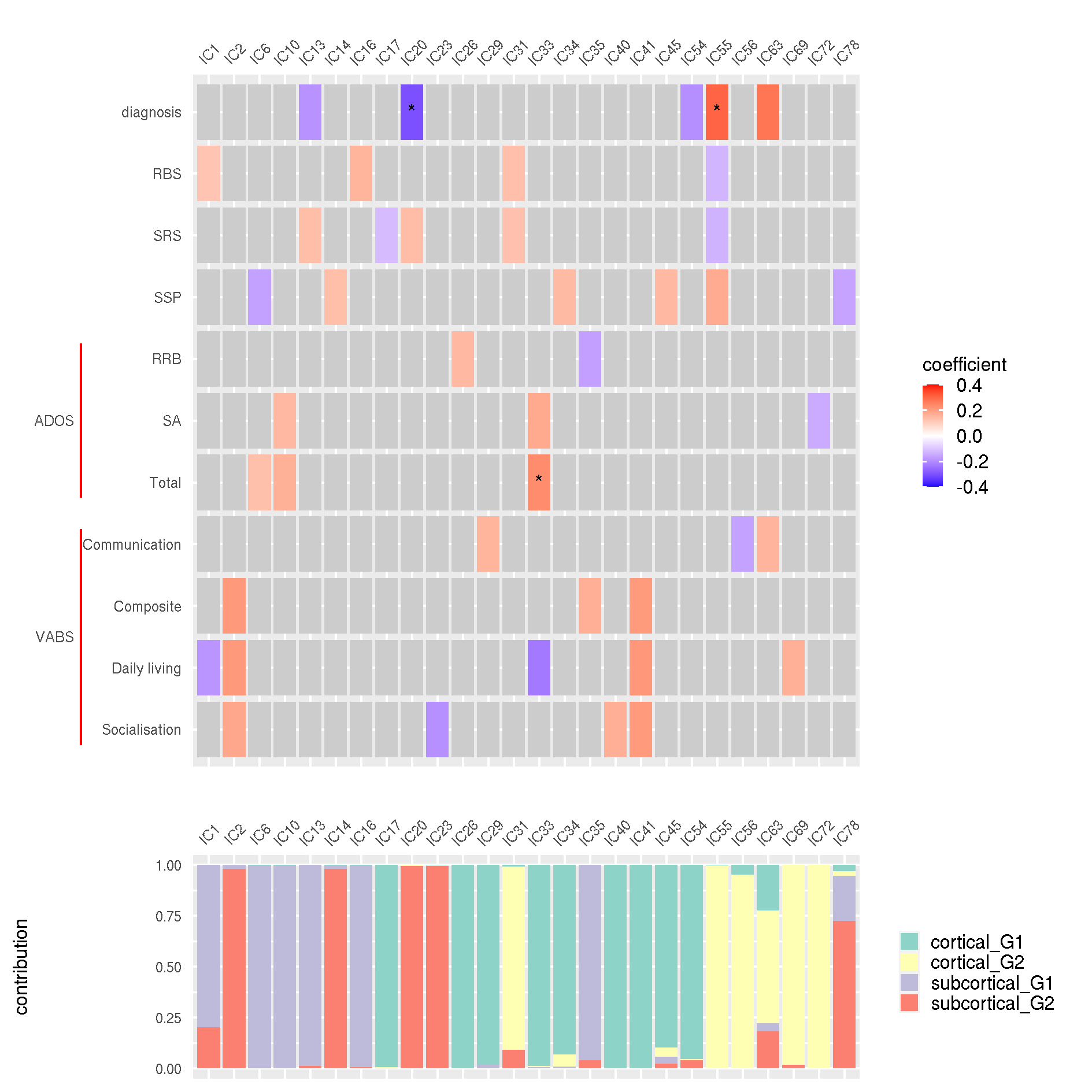


## Supplementary Figure 13 Summary of uncorrected results from functional only integration

The top figure is a heatmap of significant uncorrected results for all components with findings. All coloured tiles were nominally significant. * *p_uncorrected_* < 0.001. The bottom figure shows the corresponding feature contributions for these components. ADOS - Autism Diagnostic Observational Schedule, VABS - Vineland Adaptive Behaviours Scale, RBS - Repetitive Behaviours Scale, SRS - Social Responsiveness Scale, SSP - Short Sensory Profile, RRB - Restrictive and Repetitive Behaviours scale, SA - Social Affect scale, IC - independent component, G1/2 - gradient 1/2.

The first component, IC20, showed significant association with autism diagnosis with higher contribution of autistic participants to this component than non-autistic (Figure S12, coeff.= -0.3, *p_adj_*=0.03). There was a significant site effect (χ^2^=16.01, *p*=0.003) but no significant effect of timepoint, fsIQ or mean framewise displacement (p-values > 0.05). We found a significant diagnosis by age interaction effect (χ^2^=5.2, *p*=0.02). We did not find significant interaction effects between diagnosis and site, sex or timepoint (p-values > 0.05). The spatial maps show that the component is mostly driven by subcortical connectopic gradient 2 (99.45%), localised in the putamen of the right hemisphere (Figure S13). We checked if this component would show a correlation with components from the main analysis, to see if they capture similar variance as the main analysis components. We found that IC20 from this analysis is significantly correlated with IC3 from the main analysis (rho = -0.15, *p_perm_*=0.0002) in regards to subject contribution. Both components are driven by subcortical gradient 2 and we identified a strong correlation between their respective spatial maps of subcortical gradient 2 (*r* = 0.55).


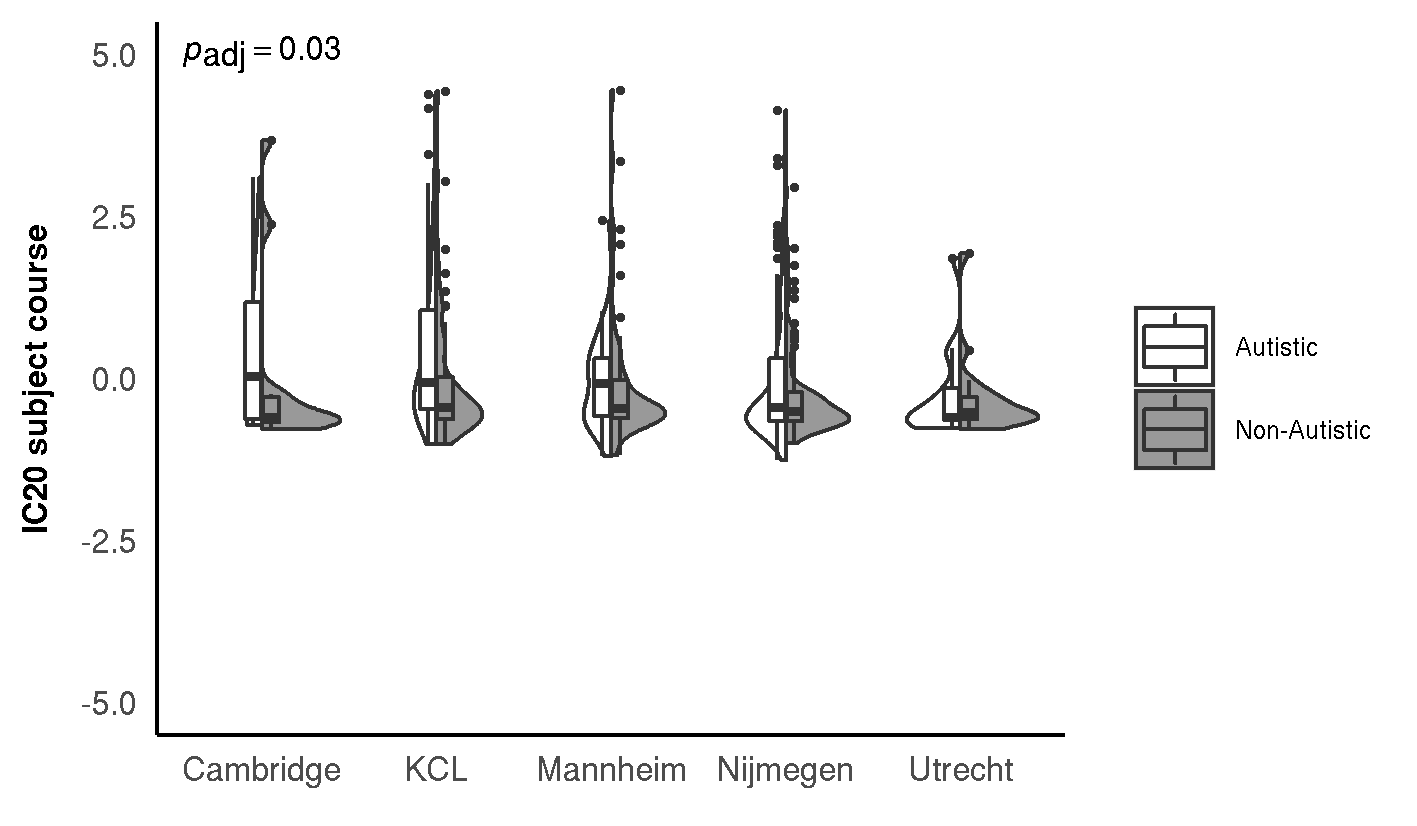


## Supplementary Figure 14 Diagnostic differences in IC20

The violin plots show the distribution of the subject courses while the boxplots indicate the first and third quartile with the median denoted with a thick horizontal line. There was a significant main effect of group. There was no significant effect of site or diagnosis-by-site interaction.

##
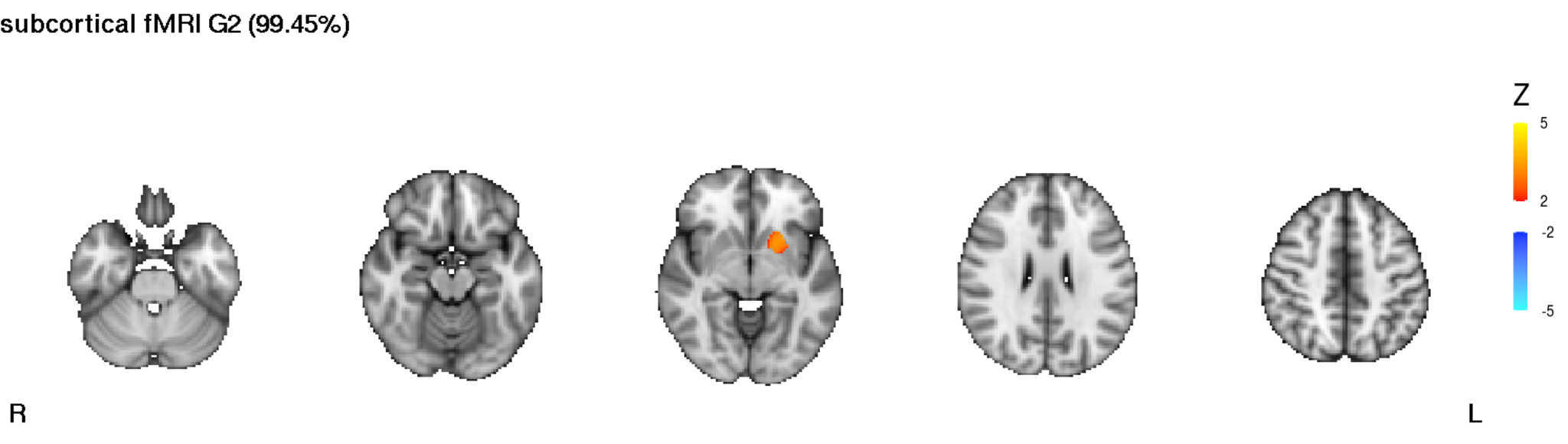


## Supplementary Figure 15 Spatial maps of IC20

The scale represents the Z-score of spatial contribution within each feature. |z| > 2 is shown. Only features contributing more than 2% are shown.

The other component significantly associated with autism diagnosis in the functional only analysis was IC55 (Figure S14, coeff.= 0.3, *p_adj_*=0.03). This association was robust to the inclusion of fsIQ and framewise displacement in the model. We found a significant diagnosis by sex effect (χ^2^=3.9, *p*=0.05). There were no significant diagnosis by site, diagnosis by timepoint, or diagnosis by sex interactions (p-values > 0.05). The spatial maps show that this component is mainly driven by cortical connectopic gradient 2 (99.87%), localised in the fusiform gyrus of the right hemisphere. The other modalities contribute <1%. As before, we checked for a correlation with components of the main analysis. We found that IC55 significantly correlated with IC62 from our main analysis (rho=0.63, *p_perm_***<0.001**) in regards to subject contribution. Both components are driven by cortical gradient 2 and the spatial maps of cortical gradient 2 correlate strongly between IC55 and IC62 (*r* = 0.96). The strength of this correlation suggests that the components capture largely the same variance in the functional domain.

##
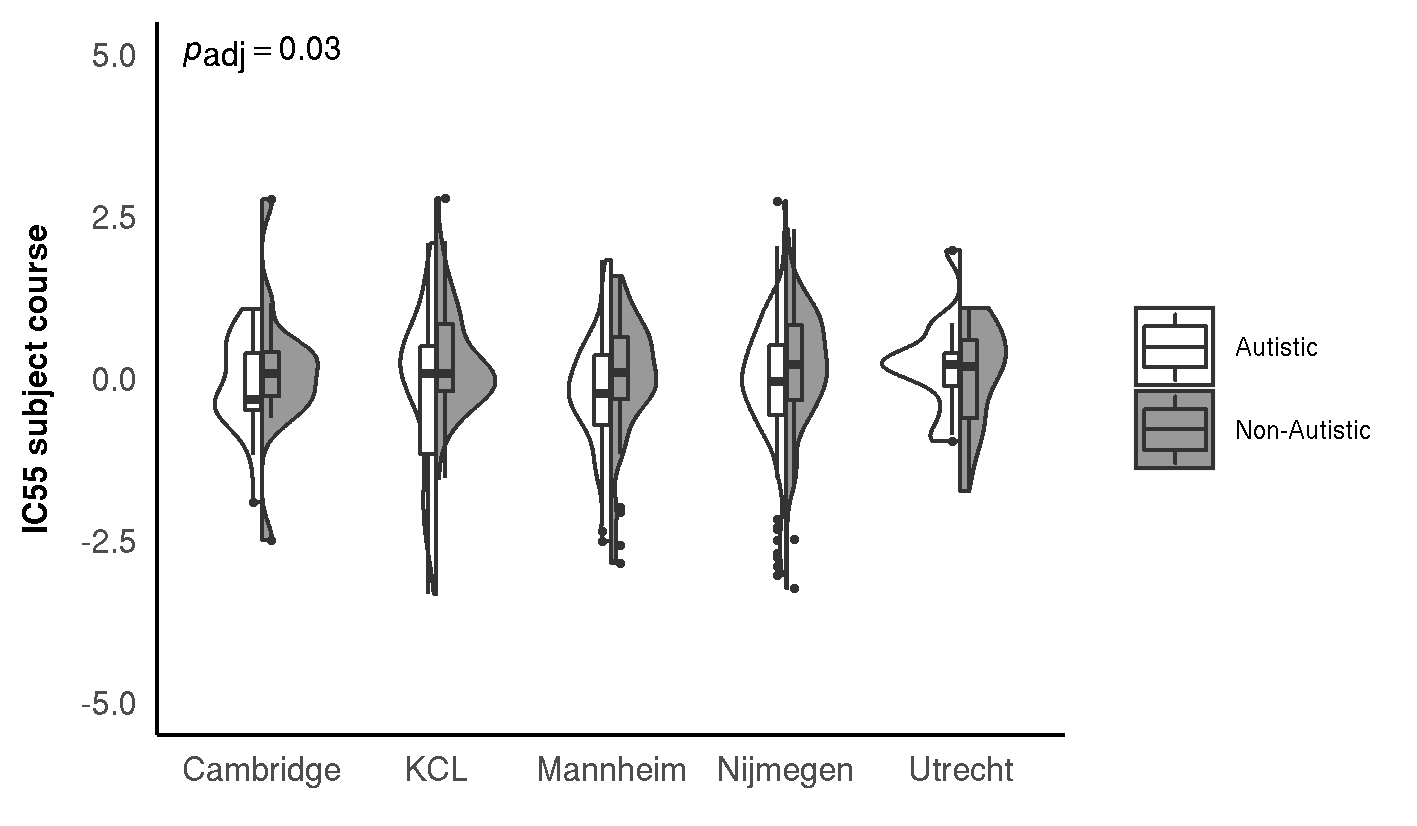
Supplementary Figure 16 Diagnostic differences in IC55

The violin plots show the distribution of the subject courses while the boxplots indicate the first and third quartile with the median denoted with a thick horizontal line. There was a significant main effect of group. There was no significant effect of site or diagnosis-by-site interaction.

##
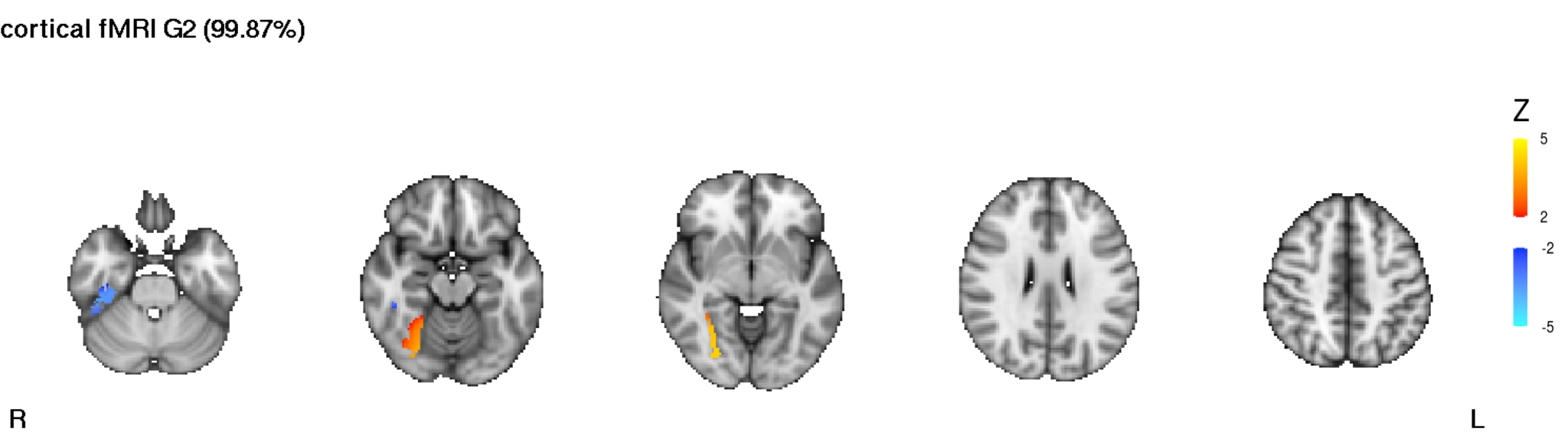


## Supplementary Figure 17 Spatial maps for IC55

Modalities contributing <1% are excluded from visualisation. The scale represents the Z-score of spatial contribution within each feature. |Z| >2 shown.

## 6.4 Comparison with Mei 2020 and Mei 2022

Given the overlap in participants with previous analyses that investigated grey matter covariation [(Mei *et al.*, 2020; n=311)](https://paperpile.com/c/DbGZMk/aTgJ) and grey-white matter covariation [(Mei *et al.*, 2022; n=315)](https://paperpile.com/c/DbGZMk/l4wr) we investigated if our main finding (IC62) captured similar variance to those reported previously (IC10 & IC14 from Mei et al. 2020; and IC58 from Mei et al. 2022). The sample from the present work shares 77% of subjects with Mei et al. 2020, and 78% of subjects with Mei et al. 2022. The subject course from IC62 did not significantly correlate with any of these previously reported components (*p_perm_* > 0.05). Thus, IC62 represents novel variance not previously captured by structural only approaches.

Our IC42 from the structural only analysis, on the other hand, was significantly related to 2 of the components reported previously (IC10 from Mei et al., 2020, rho = 0.43, *p_perm<0.001_*; IC58 from Mei et al., 2022, rho =-0.15, *p_perm_* = 0.006, Figure S16D-E).

Furthermore, the spatial maps of the VBM modality from IC42 correlated with the VBM spatial maps from both IC10 of Mei et al., 2020 (r=0.11) and IC58 from Mei et al., 2022 (r=0.11), see Figure S16A-C.

**
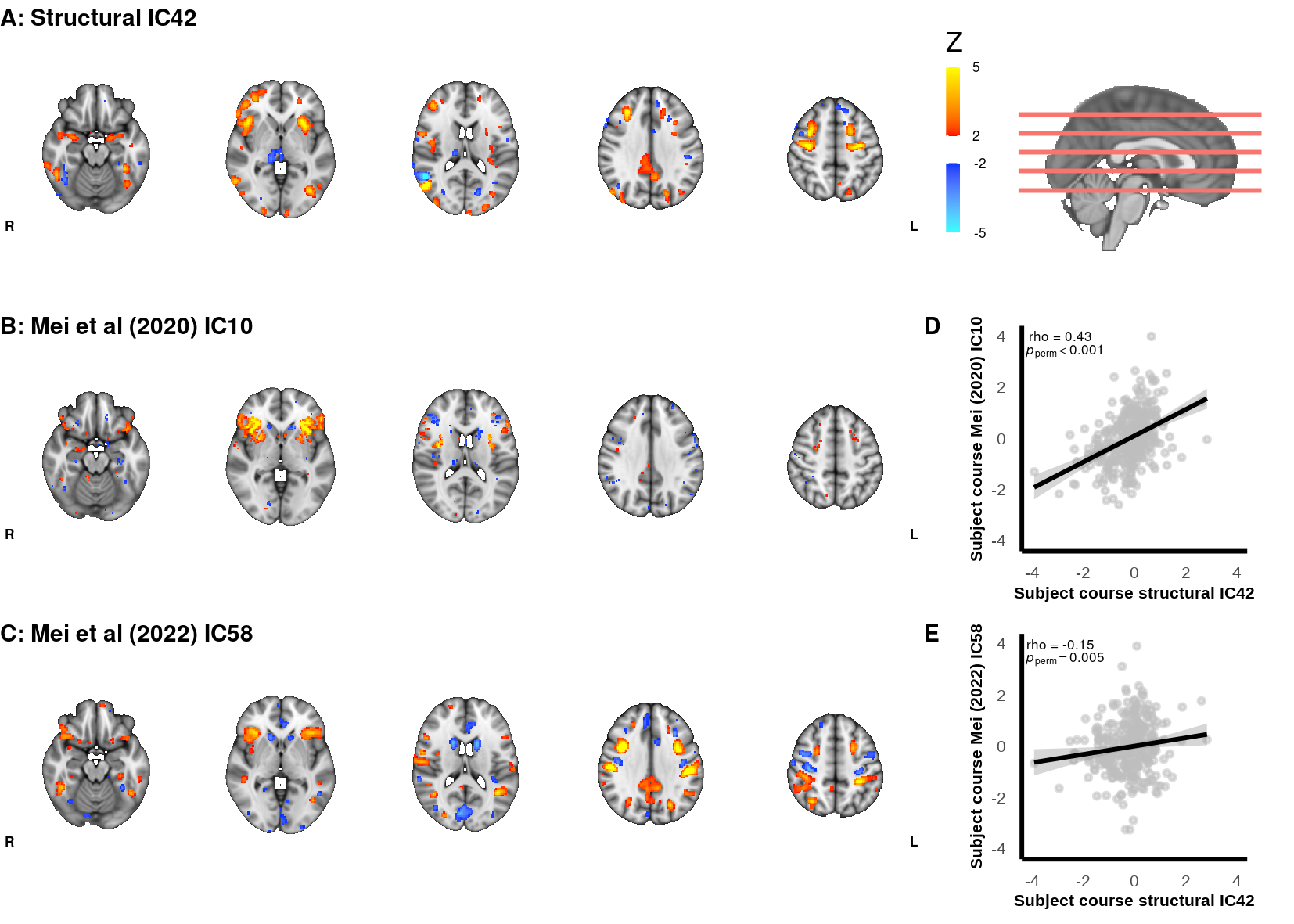
**

## Supplementary Figure 18 Comparison of structural IC42 with components from Mei et al (2020, 2022)

A-C display thresholded Z-scored spatial maps of the VBM modality for Structural IC42 (current study), IC10 (Mei et al 2020) and IC58 (Mei et al 2022), respectively. Legend associated with the spatial maps |Z| >2, axial slice locations are indicated on the sagittal slice with red lines. D and E show scatter plots of association between the subject course of structural IC42 against the subject course of IC10 (D; Mei et al 2020) and the subject course of IC58 (E; Mei et al 2022), respectively.

# References

[Andersson, J.L.R. *et al.* (2017) ‘Towards a comprehensive framework for movement and distortion correction of diffusion MR images: Within volume movement’, *NeuroImage*, 152, pp. 450–466.](http://paperpile.com/b/DbGZMk/AWmL)

[Andersson, J.L.R. and Sotiropoulos, S.N. (2016) ‘An integrated approach to correction for off-resonance effects and subject movement in diffusion MR imaging’, *NeuroImage*, 125, pp. 1063–1078.](http://paperpile.com/b/DbGZMk/iqln)

[Ashburner, J. (2007) ‘A fast diffeomorphic image registration algorithm’, *NeuroImage*, 38(1), pp. 95–113.](http://paperpile.com/b/DbGZMk/b0uT)

[Ashburner, J. and Friston, K. (1997) ‘Multimodal image coregistration and partitioning--a unified framework’, *NeuroImage*, 6(3), pp. 209–217.](http://paperpile.com/b/DbGZMk/yM2U)

[Bastiani, M. *et al.* (2019) ‘Automated quality control for within and between studies diffusion MRI data using a non-parametric framework for movement and distortion correction’, *NeuroImage*, 184, pp. 801–812.](http://paperpile.com/b/DbGZMk/sbja)

[Greve, D.N. and Fischl, B. (2009) ‘Accurate and robust brain image alignment using boundary-based registration’, *NeuroImage*, 48(1), pp. 63–72.](http://paperpile.com/b/DbGZMk/N7wh)

[Jenkinson, M. *et al.* (2002) ‘Improved optimization for the robust and accurate linear registration and motion correction of brain images’, *NeuroImage*, 17(2), pp. 825–841.](http://paperpile.com/b/DbGZMk/VZ7d)

[Kellner, E. *et al.* (2016) ‘Gibbs-ringing artifact removal based on local subvoxel-shifts’, *Magnetic resonance in medicine: official journal of the Society of Magnetic Resonance in Medicine / Society of Magnetic Resonance in Medicine*, 76(5), pp. 1574–1581.](http://paperpile.com/b/DbGZMk/jbBL)

[Koay, C.G. and Basser, P.J. (2006) ‘Analytically exact correction scheme for signal extraction from noisy magnitude MR signals’, *Journal of magnetic resonance* , 179(2), pp. 317–322.](http://paperpile.com/b/DbGZMk/GGcR)

[Kundu, P. *et al.* (2012) ‘Differentiating BOLD and non-BOLD signals in fMRI time series using multi-echo EPI’, *NeuroImage*, 60(3), pp. 1759–1770.](http://paperpile.com/b/DbGZMk/69eQ)

[Kurth, F., Luders, E. and Gaser, C. (2015) ‘Voxel-Based Morphometry’, in A.W. Toga (ed.) *Brain Mapping*. Waltham: Academic Press, pp. 345–349.](http://paperpile.com/b/DbGZMk/23gr)

[Mei, T. *et al.* (2020) ‘Gray matter covariations and core symptoms of autism: the EU-AIMS Longitudinal European Autism Project’, *Molecular autism*, 11(1), p. 86.](http://paperpile.com/b/DbGZMk/aTgJ)

[Mei, T. *et al.* (2022) ‘Autism is associated with inter-individual variations of gray and white matter morphology’, *Biological Psychiatry: Cognitive Neuroscience and Neuroimaging* [Preprint]. Available at: https://doi.org/](http://paperpile.com/b/DbGZMk/l4wr)[10.1016/j.bpsc.2022.08.011](http://dx.doi.org/10.1016/j.bpsc.2022.08.011)[.](http://paperpile.com/b/DbGZMk/l4wr)

[Perrone, D. *et al.* (2015) ‘The effect of Gibbs ringing artifacts on measures derived from diffusion MRI’, *NeuroImage*, 120, pp. 441–455.](http://paperpile.com/b/DbGZMk/hwdr)

[Pruim, R.H.R. *et al.* (2015) ‘ICA-AROMA: A robust ICA-based strategy for removing motion artifacts from fMRI data’, *NeuroImage*, 112, pp. 267–277.](http://paperpile.com/b/DbGZMk/Rk1w)

[Sotiropoulos, S.N. *et al.* (2013) ‘Effects of image reconstruction on fiber orientation mapping from multichannel diffusion MRI: reducing the noise floor using SENSE’, *Magnetic resonance in medicine: official journal of the Society of Magnetic Resonance in Medicine / Society of Magnetic Resonance in Medicine*, 70(6), pp. 1682–1689.](http://paperpile.com/b/DbGZMk/58Fy)

[Veraart, J., Fieremans, E. and Novikov, D.S. (2016) ‘Diffusion MRI noise mapping using random matrix theory’, *Magnetic resonance in medicine: official journal of the Society of Magnetic Resonance in Medicine / Society of Magnetic Resonance in Medicine*, 76(5), pp. 1582–1593.](http://paperpile.com/b/DbGZMk/SQVG)
